# A novel role for GalNAc-T2 dependent glycosylation in energy homeostasis

**DOI:** 10.1016/j.molmet.2022.101472

**Published:** 2022-03-15

**Authors:** Cristy R.C. Verzijl, Federico Oldoni, Natalia Loaiza, Justina C. Wolters, Antoine Rimbert, E. Tian, Weiming Yang, Dicky Struik, Marieke Smit, Niels J. Kloosterhuis, Amy J. Fernandez, Nadine L. Samara, Kelly G. Ten Hagen, Kruti Dalal, Aliona Chernish, Peggy McCluggage, Lawrence A. Tabak, Johan W. Jonker, Jan Albert Kuivenhoven

**Affiliations:** 1 Department of Pediatrics, University of Groningen, University Medical Center Groningen, Groningen, the Netherlands; 2 Department of Molecular Genetics, University of Texas Southwestern Medical Center, Dallas, TX, United States; 3 Nantes Université, CNRS, INSERM, l'institut du thorax, F-44000, Nantes, France; 4 Department of Biophysics and Biophysical Chemistry, Johns Hopkins University School of Medicine, Baltimore, MD, United States; 5 Structural Biochemistry Unit, National Institutes of Health, Bethesda, MD, United States; 6 Developmental Glycobiology Section, NIDCR, National Institutes of Health, Bethesda, MD, United States; 7 Section on Biological Chemistry, National Institute of Dental and Craniofacial Research, National Institutes of Health, Bethesda, MD, United States

**Keywords:** Glycosylation, Genetic disorder, Energy metabolism, Adipose tissue, Insulin signaling

## Abstract

**Objective:**

*GALNT2*, encoding polypeptide N-acetylgalactosaminyltransferase 2 (GalNAc-T2), was initially discovered as a regulator of high-density lipoprotein metabolism. GalNAc-T2 is known to exert these effects through post-translational modification, i.e., O-linked glycosylation of secreted proteins with established roles in plasma lipid metabolism. It has recently become clear that loss of *GALNT2* in rodents, cattle, nonhuman primates, and humans should be regarded as a novel congenital disorder of glycosylation that affects development and body weight. The role of GALNT2 in metabolic abnormalities other than plasma lipids, including insulin sensitivity and energy homeostasis, is poorly understood.

**Methods:**

GWAS data from the UK Biobank was used to study variation in the *GALNT2* locus beyond changes in high-density lipoprotein metabolism. Experimental data were obtained through studies in *Galnt2*^*−/−*^ mice and wild-type littermates on both control and high-fat diet.

**Results:**

First, we uncovered associations between *GALNT2* gene variation, adiposity, and body mass index in humans. In mice, we identify the insulin receptor as a novel substrate of GalNAc-T2 and demonstrate that *Galnt2*^*−/−*^ mice exhibit decreased adiposity, alterations in insulin signaling and a shift in energy substrate utilization in the inactive phase.

**Conclusions:**

This study identifies a novel role for GALNT2 in energy homeostasis, and our findings suggest that the local effects of GalNAc-T2 are mediated through posttranslational modification of the insulin receptor.

## Abbreviations

ANGPTL3: angiopoietin-like 3
APOC3: apolipoprotein C-III
BAT: brown adipose tissue
BMI: body mass index
EE: energy expenditure
EXoO: extraction of O-linked glycopeptides
GALNT2: N-acetylgalactosaminyltransferase 2, encoding GalNAc-T2
GALNT2-CDG: GALNT2-congenital disorder of glycosylation
GWAS: genome-wide association studies
HDL: high density lipoprotein
LOF: loss of function
LPL: lipoprotein lipase
NEFA: non-esterified fatty acids
PLTP: phospholipid transfer protein
RER: respiratory exchange ratio
WAT: white adipose tissue

## 1 Introduction

Genome-wide association studies (GWAS) identified *GALNT2*, encoding polypeptide N-acetylgalactosaminyltransferase 2 (GalNAc-T2), to be associated with plasma levels of high-density lipoprotein (HDL) cholesterol and triglycerides [1]. In the same study, a causal role for GalNAc-T2 in HDL metabolism was shown in mouse models [1]. The *GALNT2* gene is abundantly expressed in all tissues [2] and its translation results in GalNAc-T2, which is a glycosyltransferase that initiates mucin-type O-linked glycosylation at serine or threonine residues of target proteins. Although GalNAc-T2 modifies many target proteins, only a few have been discovered. Holleboom et al. were the first to identify apolipoprotein (apo) C-III (encoded by *APOC3*) as a potential substrate of GalNAc-T2 mediating the effect on HDL cholesterol and postprandial triglyceride levels in human heterozygous carriers of a rare variant in *GALNT2* [3]. In a subsequent study, angiopoietin-like 3 (encoded by *ANGPTL3*) was identified as another potential substrate of GalNAc-T2 [4]. Furthermore, Khetarpal et al. showed that *GALNT2* deficiency reduces HDL cholesterol levels in humans, mice and rats, and in nonhuman primates [5]. The authors confirmed that apoC-III and ANGPTL3 are substrates of GalNAc-T2 in humans and reported phospholipid transfer protein (PLTP) as an additional new substrate of GalNAc-T2 in humans and rodents [5]. While regulation of HDL metabolism has been at the center of key *GALNT2* studies, clues suggest that GalNAc-T2 plays roles beyond the regulation of plasma lipids. Recently, human *GALNT2* deficiency was presented as a syndromic disorder called *GALNT2*-congenital disorder of glycosylation (*GALNT2*-CDG). The affected individuals were shown to exhibit dysmorphic facial features, global developmental delay and intellectual disability, with mouse and rat models mimicking this phenotype [6]. Similarly, a recent study in cattle shows that homozygote carriers of splice-disrupting *GALNT2* variants display reduced body weight and stature [7]. To date, however, the association of *GALNT2* with metabolic syndrome [8], developmental delay and reduced body weight [6,7] have not been mechanistically investigated.

Kettunen et al. were the first to show an association between *GALNT2* genetic variants and metabolic syndrome [8]. Furthermore, it has been shown that *GALNT2* over-expression in pre-adipocytes improved insulin signaling, stimulated adipocyte maturation and led to enlarged mature adipocytes [9,10]. In line with these studies is a recently published review that describes several studies in adipocytes with a focus on the role of *GALNT2* in insulin sensitivity and adipose tissue homeostasis [11]. Here, we addressed the role of *GALNT2* in body weight regulation, insulin sensitivity and whole-body energy metabolism and provide new insights into the pathophysiology of murine *Galnt2* deficiency.

## 2 Materials and methods

### 2.1 Genetic association with continuous traits and biomarkers

Genetic associations with biological traits were extracted from the Pan-ancestry genetic analysis of the UK Biobank by the Pan-UK Biobank team [released June 15, 2020] [12]. In short, genetic and phenotypic data from ∼500,000 participants in the UK Biobank (https://www.ukbiob4ank.ac.uk) were used to conduct a Genome-Wide Association Study (GWAS). Genotypes were imputed from the Haplotype Reference Consortium plus UK10K & 1000 Genomes reference panels as released by UK-Biobank in March 2018. This research was conducted using the UK Biobank Resource (project ID 31063), and the use of these data is bound by all terms of usage of the UK Biobank. We made use of data related to 152 traits (listed in Table S1). Benferroni correction, based on the number of independent tests, was used to correct for multiple testing (significant threshold p = 3.29E-04 (0.05/152 traits)).

### 2.2 Generation of Galnt2-deficient mice and genotyping

Plasmids and *E. coli* strains for gene targeting were obtained from the Frederick National Laboratory for Cancer Research. The PL253 targeting vector replaces exon 7 of the *Galnt2* gene with a neo cassette flanked by loxP sites. Embryonic stem cell culture, screening, blastocyst injection and chimeric mice production were performed by the University of Cincinnati Gene Targeting and Transgenic Mouse Models Core. *Galnt2* heterozygous mice (*Galnt2*^*+/−*^) were backcrossed into the C57BL/6NHsd inbred mouse strain (The Jackson Laboratory) for over >15 generations before further analysis. Male and female *Galnt2*^*+/−*^ mice were used for crosses to obtain all three genotypes: *Galnt2*^*+/+*^, *Galnt2*^*+/−*^ and *Galnt2*^*−/−*^. The following three primers were used for PCR genotyping: P1 (5′ GGTCCTGACCTTCCTAGACAGTCACTGC 3′), P2 (5′ GCACTCTCCAAGGGCATGACAGAGC 3′) and P3 (5′ GGGGGAGGATTGGGAAGACAATAGC 3′). The wild-type allele resulted in a 1029 bp product, while the *Galnt2* mutant allele resulted in a 386 bp product.

### 2.3 Animals

Heterozygous breeding was used to obtain *Galnt2*^*−/−*^ mice and wild-type littermates. The genetically modified mice and wild-type littermates used in this study were on a 99% C57BL/6J genetic background and fed a standard chow diet (RM1; SDS Diets, Woerden, The Netherlands). Mice were housed in a light- and temperature-controlled facility with a 12-h light/dark cycle at 21 °C with free access to water and control diet consisting of 10% fat (D12450J, Research Diets) or high-fat diet consisting of 60% fat (D12492i, Research Diets). Animal experiments were performed with the approval of the National Ethics Committee for Animal Experiments of The Netherlands, in accordance with relevant guidelines and regulations (including laboratory and biosafety regulations).

### 2.4 Animal experiments

Both male and female mice between 3- and 9-week-old were group-housed with littermates, weighed weekly and fed a standard chow diet. Since the estrous cycle in female mice is known to affect metabolic regulation, 12-week-old male mice were used in all other experiments. Body weight and food intake of individually housed male mice were measured weekly. Measurements of body composition, including fat mass and lean tissue mass, were assessed at several time points (i.e., before indirect calorimetry measurements and before sacrifice) in non-anesthetized mice using nuclear magnetic resonance (NMR) MiniSpec (MiniSpec LF90 BCA-analyzer, Bruker). Blood was collected via retro-orbital bleeding under isofluorane anesthesia in citrate-EDTA tubes, and plasma was isolated by centrifugation at 1000×*g* for 10 min at 4 °C and stored at −80 °C until further analysis. Mice were sacrificed under isofluorane anesthesia after a 4- to 6-h fasting period in the morning, after which blood was drawn by cardiac puncture followed by cervical dislocation. Fat pads, liver and quadriceps were weighed, and tissues for mRNA and protein expression analysis were snap-frozen in liquid nitrogen and stored at −80 °C until further analysis.

### 2.5 Glucose homeostasis

Glucose homeostasis was assessed by glucose and insulin tolerance tests (GTT, ITT). For glucose tolerance, mice were fasted for 6 h after the dark phase. Mice received an intraperitoneal injection of glucose solution of 2g dextrose/kg of body weight (Sigma–Aldrich, #G6152). Blood glucose levels were measured using a glucometer (Accu-Check Performa, Roche) before and 15, 30, 60, 90, and 120 min post injection. To assess insulin tolerance, mice were fasted for 6 h after the dark phase. Mice received an intraperitoneal injection containing insulin at a dose of 0.75 units/kg of body weight (insulin aspart, NovoRapid). Blood glucose levels were measured before and 15, 30, 60, 90, and 120 min post injection.

### 2.6 Indirect calorimetry

Indirect calorimetry was performed using fully automated metabolic cages (LabMaster, TSE systems). After at least 24 h of acclimatization, O_2_ consumption (VO_2_), CO_2_ production (VCO_2_) and caloric intake were measured for at least three consecutive days. The respiratory exchange ratio (RER) (VCO_2_/VO_2_) and energy expenditure (EE) (((3.941 ∗ VO_2_) + (1.106 ∗ VCO_2_) ∗ 1.44) were calculated from the VO_2_ and VCO_2_. Glucose oxidation was calculated using the formula (((4.585 ∗ VCO_2_) – (3.226 ∗ VO_2_)) ∗ 4) and fat oxidation was calculated using (((1.695 ∗ VO_2_) – (1.701 ∗ VCO_2_)) ∗ 9). *CalR* was used for generalized linear model and ANOVA analysis [13]. In total, four independent indirect calorimetry experiments were performed in different cohorts with 7–8 mice per genotype.

### 2.7 In vivo lipolysis assay

To assess insulin-mediated suppression of lipolysis, mice were fasted for 6 h after the dark phase. Hereafter, mice received an intraperitoneal injection containing insulin (0.75 units/kg of body weight). Blood was collected via the tail vein before and 15 and 30 min post injection. Plasma was obtained by centrifugation at 1000×*g* for 10 min at 4 °C and NEFA levels were measured as described below.

### 2.8 Ex vivo lipolysis assay

For *ex vivo* lipolysis determination, visceral white adipose tissue pieces of 20 mg were incubated at 37 °C in 200 μL Krebs Ringer buffer (12 mM HEPES, 4.9 mM KCl, 121 mM NaCl, 1.2 mM MgSO_4,_ and 0.33 mM CaCl_2_) containing 3.5% fatty-acid free BSA. Buffer samples were collected after 0, 1, 2, 3, and 4 h. Glycerol and free fatty acids were measured according to the manufacturer's protocol using the Free Glycerol Colorimetric Assay Kit II (Biovision, #K634-100) and Free Fatty Acid Quantification Colorimetric/Fluorometric Kit (Biovision, #K612-100), respectively.

### 2.9 Ex vivo insulin signaling

*Ex vivo* insulin signaling was assessed by incubating small pieces of visceral white adipose tissue in Krebs Ringer buffer (as described above) supplemented with 0.5U/mL insulin (50–100 mg per condition in 12-well plates). Medium was discarded and tissues were harvested after 0, 5 and 15 min by a short spin at 4000×*g* at 4 °C and lysed using ice-cold NP40 buffer (0.1% Nonidet P-40 [NP-40], 0.4 M NaCl, 10 mM Tris–HCl [pH 8.0], 1 mM EDTA) supplemented with protease and phosphatase inhibitors (Roche).

### 2.10 Oral fat tolerance test

Mice were fasted for 4 h prior to receiving an oral fat load using olive oil (100 μL per 10 g of body weight). Blood samples were collected from the tail vein before the oral fat load and after 60, 90, 120, 180, and 240 min following gavage. After centrifugation at 1000×*g* for 10 min at 4 °C, plasma was collected and used for triglyceride measurements as described below.

### 2.11 Plasma lipids measurement

Plasma levels of total cholesterol (TC) were determined using a colorimetric assay (Roche, #11489232) with a Cholesterol Standard (Diasys Diagnostic Systems GmbH, #113009910030). Plasma triglycerides were measured using a colorimetric assay (Roche, #1187771) using a Precimat glycerol standard (Roche, #16658800). Non-esterified fatty acids (NEFAs) were measured by colorimetric assay (Diasys Diagnostics, #157819910935) using a NEFA standard (DiaSys Diagnostics, #157809910065). All measurements were performed as described in the manufacturer's protocol.

### 2.12 Fast-performance liquid chromatography (FPLC)

Total cholesterol and triglyceride content of the major lipoprotein classes (VLDL, LDL and HDL) were measured using FPLC analysis. In short, the system consisted of a PU-4180 RHPLC pump and a UV-4075 UV-Vis detector (Jasco). Plasma samples of each experimental group of mice were pooled, diluted in PBS and loaded onto a Superose® Increase 10/300 GL column (GE Healthcare) for separation of lipoproteins at a flow rate of 0.31 mL/min. A second flow was used to add the cholesterol (Roche, #1489232) enzymatic reagent at a flow rate of 0.10 mL/min.

### 2.13 Hepatic lipid extraction and measurement of hepatic phospholipids, cholesterol, free cholesterol and triglycerides

Lipid extraction was performed on liver homogenates (15% w/v in PBS) following the Bligh and Dyer method [14]. In brief, 100 μL of liver homogenate was added to 700 μL of H_2_O and subsequently mixed with 3 mL chloroform/methanol (1:2 v/v). After 30 min of incubation, 1.2 mL of H_2_O and 1 mL of chloroform were added, mixed and centrifuged at 500×*g* for 10 min at room temperature. After transferring the organic layer to a new glass tube, the solvent was evaporated with nitrogen. Dried lipids were dissolved in 1 mL chloroform. For phospholipids, 75 μL of the lipids dissolved in chloroform was evaporated, and dried lipids were dissolved in 400 μL 70% HClO_4_ then boiled for 60 min at 160 °C after which 4.5 mL phosphate reagent and 500 μL ascorbic acid (10% w/v) were added. Samples were boiled, allowed to cool down to room temperature and absorption was measured at 750 nm. For the quantification of cholesterol (DiaSys Diagnostics, #113009910026), free cholesterol (Spinreact, #41035) and triglyceride (DiaSys Diagnostics, #57109910917) content, 400 μL of the lipids dissolved in chloroform was evaporated and dried lipids were solubilized in 500 μL 2% Triton-X-100 in chloroform. The solvent was evaporated with nitrogen, and the Triton-X-100 dissolved lipids were solubilized in 500 μL H_2_O.

### 2.14 Histology and H&E staining

Tissues were fixed in 4% (w/v) phosphate-buffered formalin, embedded in paraffin and sectioned at 4 μm. Sections of visceral WAT were stained with hematoxylin and eosin (H&E) according to standard protocols. Slides were scanned with the Hamamatsu slide scanner and quantified using ImageJ [15].

### 2.15 Determination of hormone and ketone bodies concentrations

Plasma insulin levels were measured using the Ultra-Sensitive Rat Insulin ELISA Kit (CrystalChem, #90060) according to the manufacturer's protocol using Mouse insulin as a standard (CrystalChem #90020). Glucagon was measured using the Mouse Glucagon ELISA kit (Chrystal Chem, USA, #81518). Levels of catecholamines, corticosterone and their metabolites were determined by XLC-MS/MS (as described previously in [16,17]). Plasma β-hydroxybutyrate was measured in 4-h fasted mice using the FreeStyle precision β-Ketone strips (Abbott Diabetes Care, #06905386) and an Optimum Xceed meter (Abbott Diabetes Case, #16648).

### 2.16 Expression and purification of GalNAc-T2

The gene expressing N-terminal His-tagged human GalNAc-T2 (hGalNAc-T2, aa 75–571) was introduced into the *Pichia pastoris* his4Δ strain Bg12 (BioGrammatics) by linearizing the expression vector pKN55-6XHis-TEV-hGalNac-T2 [18] with PmeI (New England Biolabs) followed by electroporation (Biorad). The cells were grown to an OD_600_ ∼ 10 at 30 °C in MGY-case media (1% yeast extract, 1% casamino acids, 1% yeast nitrogen base with ammonium sulfate, without amino acids, 1% glycerol) and cleared by centrifugation at 2000×*g* for 10 min. Cell pellets were resuspended in 1/5 volume of MMY-case media (1% glycerol is replaced with 2% methanol) for induction at 20 °C for 24 h. The supernatant was cleared by centrifugation at 2000×*g* for 10 min and filtered, and its pH was adjusted by adding 50 mM Tris pH 7.5 and 10 mM β-mercaptoethanol. To purify His_6_-TEV-hGalNAc-T2, the supernatant was applied to a 5 mL HisTrap column (GE Healthcare) pre-equilibrated with buffer containing 250 mM NaCl, 10 mM βME, 25 mM Tris, pH 7.5, and eluted with equilibration buffer containing 500 mM imidazole over 10 CV. The peak fractions were pooled and incubated with His_6_-TEV protease overnight at 4 °C while dialyzing into equilibration buffer containing 25 mM imidazole. To separate GalNAc-T2 from the cleaved His_6_-Tag and His_6_-TEV, the sample was manually loaded onto a 1 mL HisTrap column (GE Healthcare), pre-equilibrated with dialysis buffer, and the column was washed with 4 mL of dialysis buffer. The flow-through and wash containing pure hGalNAc-T2 were pooled, and 15% glycerol was added before aliquoting and snap freezing in LN_2_ for storage at −80 °C.

### 2.17 GalNAc-T2 activity assay

The ISOGlyP (Isoform Specific O-Glycosylation Prediction, http://isoglyp.utep.edu/index.php) server was initially used to predict the sites in the insulin receptor that are preferentially glycosylated by GalNAc-T2. The selected insulin receptor peptides were synthesized and purified by Anaspec. Reactions were initiated by adding 500 μM peptide, 7.3 μM C^14^-UDP-GalNAc, 44 μM UDP-GalNAc, 10 mM MnCl_2_, 40 mM cacodylate pH 6.5 and 40 mM β-mercaptoethanol to 0.4 μM GalNAc-T2 at a final volume of 25 μL. Reactions were carried out at 37 °C for 30 min in triplicate and stopped by adding 40 mM EDTA. Reaction products were purified by loading on to anion exchange spin columns containing 100 μL of glass beads (Sigma) and 500 μL of AG 1X-8 resin (Biorad) followed by centrifugation at 1800×*g*. The resin was washed 3 times by adding 100 μL of H_2_O and centrifugation at 1800×*g*, and the flow through and wash were pooled and analyzed by liquid scintillation counting. The background activity (the activity measured in absence of synthetic peptides) was subtracted from the experimental values. The resulting experimental values were averaged, and the standard error was calculated for each reaction.

### 2.18 O-glycosylation site-specific analysis of INSR in Galnt2^−/−^ and wild type livers

Site-containing O-glycopeptides were enriched using the EXoO method [19]. The O-glycopeptides (∼80 μg) were fractionated to 24 fractions followed by LC-MS/MS analysis using the Orbitrap Fusion™ Lumos™ Tribrid™ Mass Spectrometer with the HCD-pd-EThcD fragmentation method [20]. MSfragger-Glyco [21] and pGlyco3 [22] were used for the identification of site-specific O-glycopeptides. A total number of 1327 and 1573 glycopeptides containing 1061 and 1212 O-glycosites were identified from livers obtained from wild-type and *Galnt2*^*−/−*^ mice, respectively. The total peptide-to-spectrum matches (PSM) were 11,721 and 12,019 for livers derived from wild-type and *Galnt2*^*−/−*^ mice, so the PSM ratio between samples obtained from wild-type and *Galnt2*^*−/−*^ mice was 0.975, suggesting that data normalization was unnecessary. Furthermore, proteomic analysis of the paired wild-type and *Galnt2*^*−/−*^ samples using TMTpro™ 16plex Label Reagent revealed equivalent insulin receptor protein levels. In addition, the median number of PSM of peptides was both three in the wild type and *Galnt2*^*−/−*^ samples providing further support that normalization was not required.

### 2.19 Quantitative real-time PCR (qPCR)

The qPCR was used to determine the level of *Galnts* gene expression in livers from wild-type and *Galnt2*^*−/−*^ mice. DNase-free RNA was isolated using the PureLink® RNA Mini Isolation Kit (Ambion). cDNA synthesis was performed using the iScript cDNA Synthesis Kit (Bio-Rad). PCR primers used were published previously [23] using Beacon Designer software (BioRad). The qPCR was performed on a CFX96 real time PCR thermocycler (Bio-Rad) using the SYBR-Green PCR Master Mix (Bio-Rad). The qPCR was performed in triplicate, and four independent experiments were performed. Gene expression levels were normalized to *29S* rRNA and displayed as relative expression levels.

### 2.20 Western blot analysis

Tissue homogenates were obtained using NP40 buffer (0.1% Nonidet P-40 [NP-40], 0.4 M NaCl, 10 mM Tris–HCl [pH 8.0], 1 mM EDTA) supplemented with protease and phosphatase inhibitors (Roche). Protein concentration was determined using the Bradford assay (Bio-Rad). A total of 15–20 μg of protein per sample was separated using SDS-PAGE and transferred to PVDF Transfer Membrane (Amersham™ Hybond™ –P, GE Healthcare; RPN303F). Membranes were blocked in 5% BSA in Tris-buffered saline with 0.01% Tween 20 (Millipore Sigma) and incubated with the indicated antibodies. Proteins were visualized using a ChemiDoc XRS + System using Image Lab software version 5.2.1 (Bio-Rad).

### 2.21 Antibodies

The following primary antibodies were used for Western blotting: rabbit-anti-Insulin receptor β (Santa Cruz, #711), rabbit-anti-phospho Akt (Ser473) (Cell Signaling, #4060), rabbit-anti-Akt (Cell Signaling, #9272), rabbit-anti Gsk3β (Cell Signaling, #9315), rabbit-anti-phospho Gsk3β Ser9 (Cell Signaling, #5558), rabbit-anti FoxO1 (Cell signaling, #2880), rabbit-anti-phospho FoxO1 Ser256 (Cell Signaling, #9461), rabbit-anti p70 S6 kinase (Cell Signaling, #2708), mouse-anti-phospho p70 S6 kinase Thr389 (Cell Signaling, #9206), mouse-anti S6 (Cell Signaling, #2317), rabbit-anti-phospho S6 Ser235/236 (Cell signaling, #4856), rabbit-anti 4E-BP1 (Cell Signaling, #9644), rabbit-anti-phospho 4E-BP Thr37/46 (Cell Signaling, #9459), mouse-anti-Gapdh (Abcam #ab8245), rabbit-anti-phospho HSL Ser660 (Cell Signaling, #4126), rabbit-anti-phospho HSL Ser563 (Cell Signaling, #4139), rabbit-anti-HSL (Cell Signaling, #4107), rabbit-anti-PLIN1 (Cell Signaling, #9349) and rabbit-anti-CD36 (Cell Signaling #14347). Secondary antibodies used for western blotting were goat-anti-rabbit IgG-HRP conjugate (Biorad, #1706515) and goat-anti-mouse IgG-HRP conjugate (Biorad, #1706516).

### 2.22 Targeted proteomics

Targeted proteomics was used to quantify proteins in plasma or mitochondrial proteins in homogenized skeletal muscles tissues via isotopically labeled peptide standards (containing ^13^C-labeled lysines or arginines) derived from synthetic protein concatamers (QconCAT) (PolyQuant GmbH, Germany) as described for the lipid metabolic proteins [24] and the mitochondrial proteins [25]. For the detection and quantification of Galnt2 in liver and visceral WAT, the isotopically labeled synthetic peptide NVPYGNIQSR (containing a ^13^C^15^N-labeled arginine, AQUA Basic, Thermo Scientific) was used. For the plasma protein targets, the presented quantification results in this paper were limited to the following subset of peptides/proteins: AVEPQLEDDER and GVQIPLPEGINFVR for phospholipid transfer protein (PLTP), LESLLEEK and IYAIVQQSNYILR for angiopoietin-related protein 3 (ANGPTL3) and TVQDALSSVQESDIAVVAR for apolipoprotein C-III (APOC3). Plasma adiponectin (ADIPOQ) levels were measured using the following peptides: IFYNQQNHYDGSTGK and VTVPNVPIR. These peptides were selected not only for their optimal detection properties and uniqueness of the sequence toward the intended protein targets but also to avoid inclusion of previously reported glycosylation sides as reported in UniProt (www.uniprot.org); the determined protein concentrations are therefore not affected by potential changes in glycosylation.

### 2.23 Statistical analysis

Statistical analysis was performed using the GraphPad Prism 9.0 software package (GraphPad Software, San Diego, Ca, USA). Significance was determined using an unpaired t-test when comparing two groups. Longitudinal data were analyzed using two-way ANOVA with repeated measures, and the area under the curve was calculated and plotted in a separate figure. *CalR* was used for statistics of indirect calorimetry experiments [13]. All values are given as means ± SEM, and a P-value of less than 0.05 was considered statistically significant. Significance was indicated as ∗ P < 0.05, ∗∗ P < 0.01, ∗∗∗ P < 0.001, ∗∗∗∗ P < 0.0001.

## 3 Results

### 3.1 GALNT2 is associated with changes in plasma lipids and body fat

**Figure 1:**
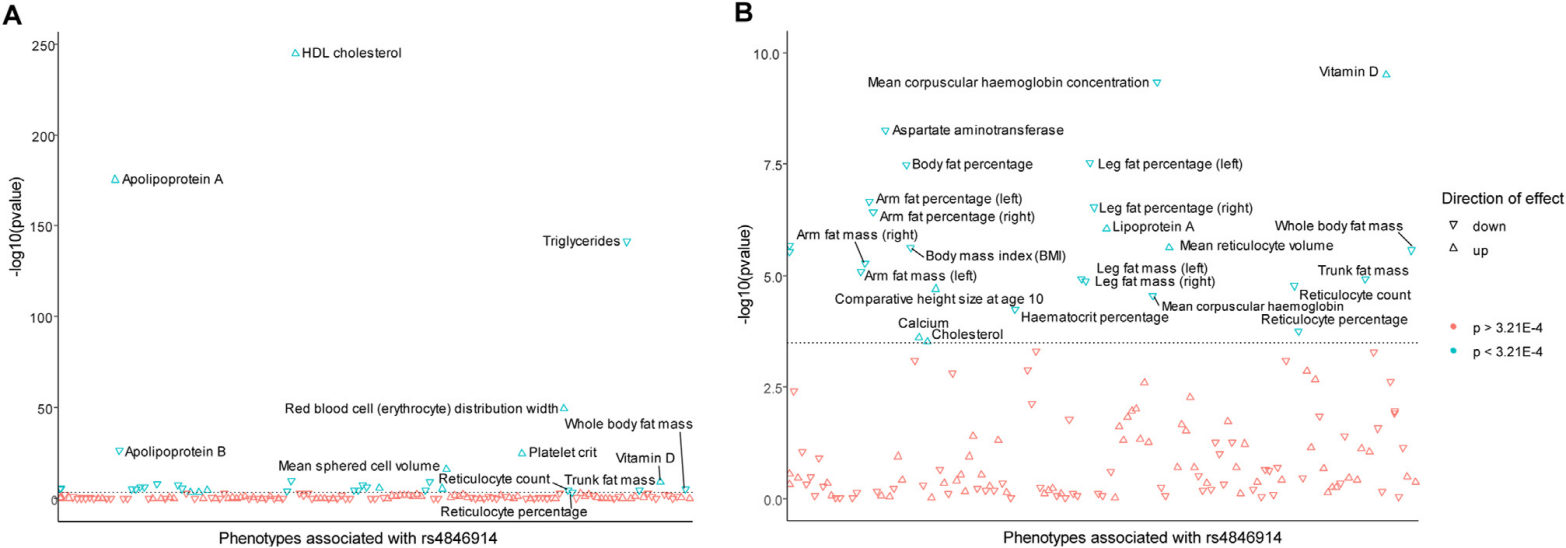
***GALNT2* SNP rs4846914 is associated with plasma lipids and body fat percentage.** Phenotypes associated with rs4846914 versus –log10 (P value) of traits and biomarkers of rs4846914 carriers. (A) Overall phenotype versus –log10 (P value) of traits and biomarkers of rs4846914 carriers. (**B**) Phenotype versus –log10 (P value) ranging from 0.0 to 10.0. Shown in (**A**) and (**B**) are the direction of effects (beta_meta) and p-value threshold and a dotted line indicating the significant threshold for multiple testing of p = 3.21E-04.

By using the top-associated single nucleotide polymorphism (SNP) from previous studies [1,5] (rs4846914) as a genetic tool, we tested the association of *GALNT2* in a selection of 152 traits (see Supplementary Table 1) measured in the UK Biobank. Rs4846914 is a common variant, with an A allele frequency of 61% in the European (non-Finnish) population (https://gnomad.broadinstitute.org) and 59% in the UK Biobank cohort. In addition to its very strong association with HDL cholesterol (p = 2.72E-245) and triglyceride (p = 4.06E-142) levels (Figure 1A), we found that rs4846914 is also significantly associated with body mass index (BMI), body fat percentage and whole-body fat mass (p-values < 2.68E-06) (Figure 1B), indicating a potential role for *GALNT2* in the regulation of body weight and whole-body energy homeostasis.

### 3.2 *Galnt2*^*−/−*^ mice present with reduced plasma lipids

**Supplementary Figure 1:**
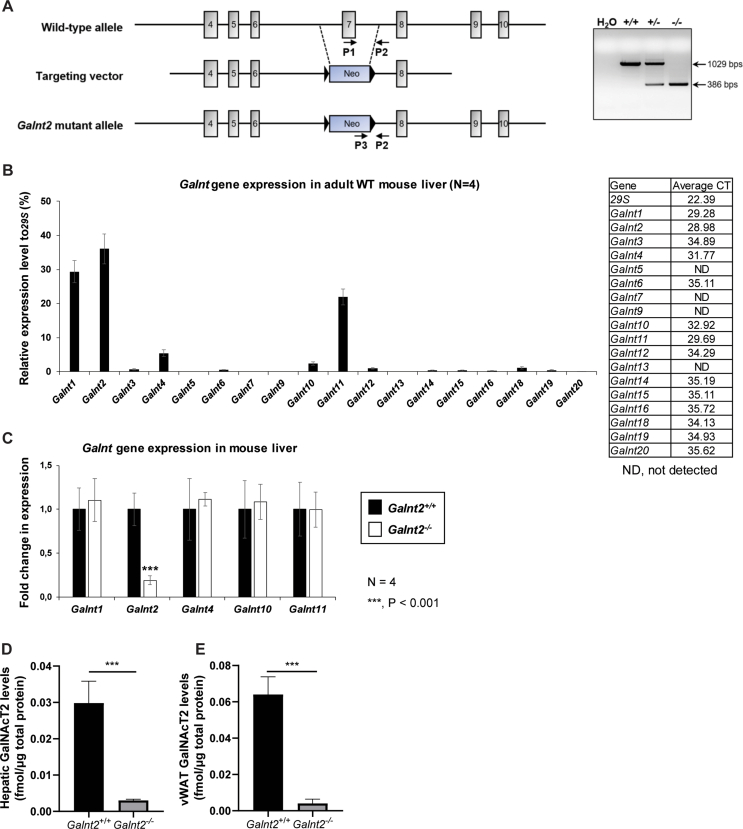
**Generation of *Galnt2* deficient mice, gene expression levels of transferases and resulting GalNAc-T2 protein levels in liver and visceral WAT.** (**A**) The gene targeting construct deletes exon 7 of the *Galnt2* gene, which encodes a highly conserved region of the catalytic domain of the *Galnt2* enzyme. Arrows show positions of the primers used for PCR genotyping (P1: 5′ GGTCCTGACCTTCCTAGACAGTCACTGC 3′, P2: 5′ GCACTCTCCAAGGGCATGACAGAGC 3′) and P3: 5′ GGGGGAGGATTGGGAAGACAATAGC 3′)). On the right is an agarose gel of PCR products from wild-type (+/+) (1029 bp band), *Galnt2*^*+/−*^ heterozygotes (1029 and 386 bp bands) and *Galnt2*^*−/−*^ mutants (386 bp band). (**B**) Gene expression levels of *Galnts* in wild-type livers with average CT levels per *Galnt* on the right side (n = 4). (**C**) *Galnt* gene expression levels abundance in livers of wild-type versus *Galnt2*^*−/−*^ mice (n = 4). (**D**) GalNAc-T2 protein levels in liver and (**E**) visceral WAT as determined using targeted proteomics (n = 5–6). Data are presented as mean values ±SEM with ∗∗∗p < 0.001, ND = not detected

To gain insight into the association between *GALNT2* and metabolic-related traits, we generated a *Galnt2*^*−/−*^ mouse model (Fig. S1A). We observed a dramatic reduction of hepatic *Galnt2* gene expression, with no effects on expression of other abundant GalNAc-transferase genes, i.e., *Galnt1*, *Galnt4*, *Galnt10* and *Galnt11* (Figs. S1B and C). This indicates that there is no compensatory upregulation at the mRNA level by other members of the GalNAc-transferase family. Using targeted proteomics, we confirmed the absence of GalNAc-T2 protein levels in liver and visceral white adipose tissue (WAT) where *Galnt2* is normally abundantly expressed (Figs. S1D and E) [26,27].

**Supplementary Figure 2:**
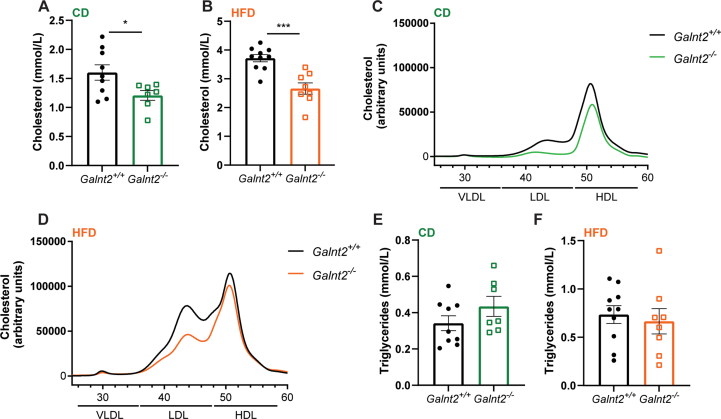
**Plasma cholesterol levels are decreased in *Galnt2***^***−/−***^**mice**. Four-hour fasted plasma cholesterol levels after (**A**) control diet and (**B**) HFD. Cholesterol levels after (**C**) control or (**D**) HFD in pooled plasma of *Galnt2*^*−/−*^ mice and wild-type littermates fractionated by FPLC. Four-hour fasted plasma triglyceride levels after (**E**) control diet and (**F**) HFD. Data presented as mean values ±SEM with ∗p < 0.05, ∗∗∗p < 0.001 (n = 7–9)

*Galnt2*^*−/−*^ mice have reduced fasting plasma cholesterol levels, on both control and high-fat diet (HFD), compared with wild-type littermates due to a reduction of cholesterol in both HDL and low-density lipoproteins (LDL) (Figs. S2A–D) as described previously [4]. We furthermore observed a trend for decreased fasting plasma triglyceride levels when *Galnt2*^*−/−*^ mice were fed a control diet but not when fed a HFD (Figs. S2E and F).

### 3.3 *Galnt2* deficient mice are smaller and gain less weight on control and high-fat diets

**Figure 2:**
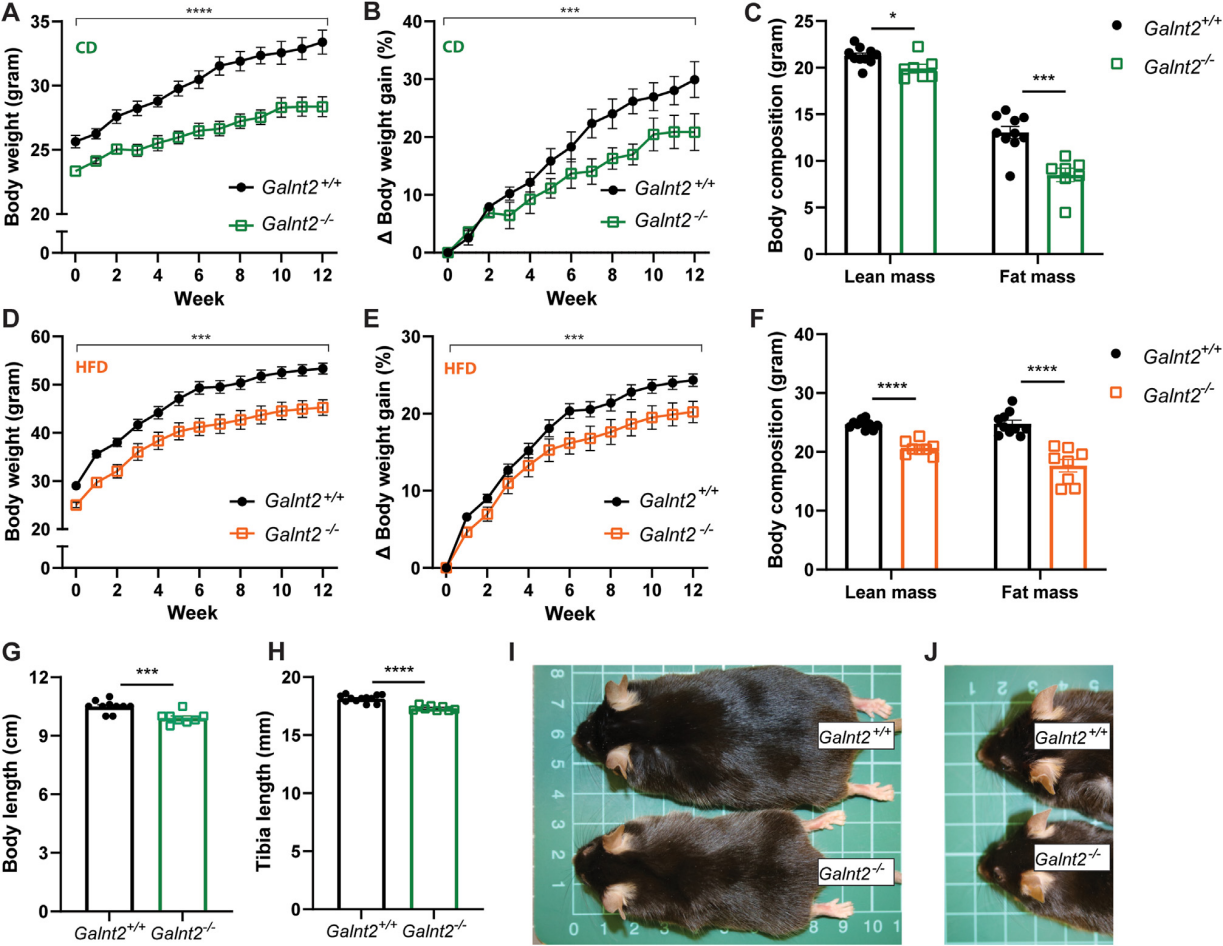
***Galnt2***^***−/−***^**mice are smaller and exhibit a GALNT2-CDG like phenotype.** Male *Galnt2*^*−/−*^ mice and wild-type littermates were followed on a control diet (CD) whereby (**A**) body weight, (**B**) body weight gain and (**C**) body composition was monitored (n = 8–9). Male *Galnt2*^*−/−*^ mice and wild-type littermates at 12 weeks of age were followed on a high-fat diet (HFD) whereby (**D**) body weight, (**E**) body weight gain and (**F**) body composition was monitored (n = 9–10). (**G**) Body length (n = 8–10) and (**H**) tibia length (n = 8–10) along with photographic representations of (**I**) the body and (**J**) head of a *Galnt2*^*−/−*^ mouse and wild-type littermate (*Galnt2*^*+/+*^). The experiments on control diet were repeated 3 times (**A-C**), and on HFD 2 times (**D-F**), (**G-J**) was determined in one cohort, but the effect was also observed in all other cohorts. Data are presented as mean values ± SEM with ∗p < 0.05, ∗∗p < 0.01, ∗∗∗p < 0.001, ∗∗∗∗p < 0.0001.

**Supplementary Figure 3:**
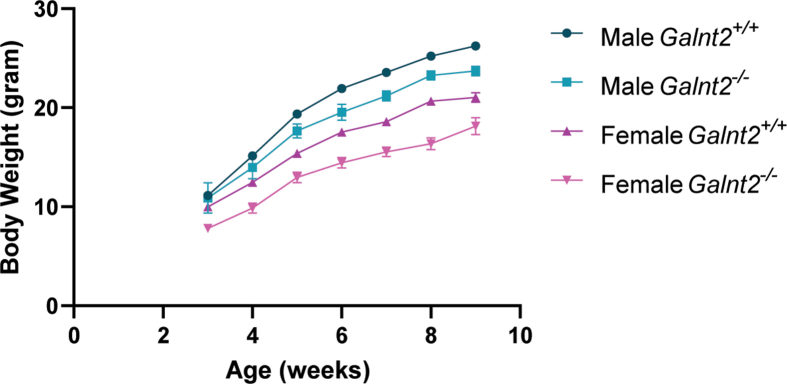
**Pup weight of male and female Galnt2**^**−/−**^**mice and wild-type littermates**. Body weight of male and female mice aged three to nine weeks

Corroborating results with another *Galnt2*^*−/−*^ mouse line [6], we observed a similar embryonic lethality following heterozygous breeding, while homozygous breeding was unsuccessful (data not shown). Early in life—in fact, from the earliest point that we could measure, i.e., from three to nine weeks of age—a reduction in body weight was observed in both surviving male and female *Galnt2*^*−/−*^ mice compared with wild-type littermates (Fig. S3). Given the similarity of this effect in both genders, further studies were performed in male mice only. On a control diet, adult male *Galnt2*^*−/−*^ mice of 10–12 weeks of age remained smaller and gained less weight compared with control mice over a period of 12 weeks (Figure 2A,B). This was characterized by a significant reduction in both lean and fat mass (Figure 2C). When adult male *Galnt2*^*−/−*^ mice of 10–12 weeks of age were challenged with a HFD for 12 weeks, we observed a consistently reduced body weight and body weight gain compared with controls (Figure 2D,E). This was again characterized by reductions in lean mass and fat mass (Figure 2F) that were more pronounced compared with *Galnt2*^*−/−*^ mice fed the control diet (Figure 2F versus Figure 2C). In addition to decreased body weight gain, *Galnt2*^*−/−*^ mice also displayed reduced body, tibia and snout length (Figure 2G–J). The reduced body weight and stature phenotype have been previously observed in *Galnt2* deficient mice, as well as in humans and dairy cattle [6,7].

In line with our genetic observations in humans, our mouse data show that *Galnt2* plays a role in whole-body energy homeostasis: *Galnt2*^*−/−*^ mice remain smaller and gain less weight on both control and HFD compared with wild-type littermates, and this can be explained by reduction in lean and fat mass.

### 3.4 *Galnt2* deficiency is associated with reduced visceral WAT mass

**Figure 3:**
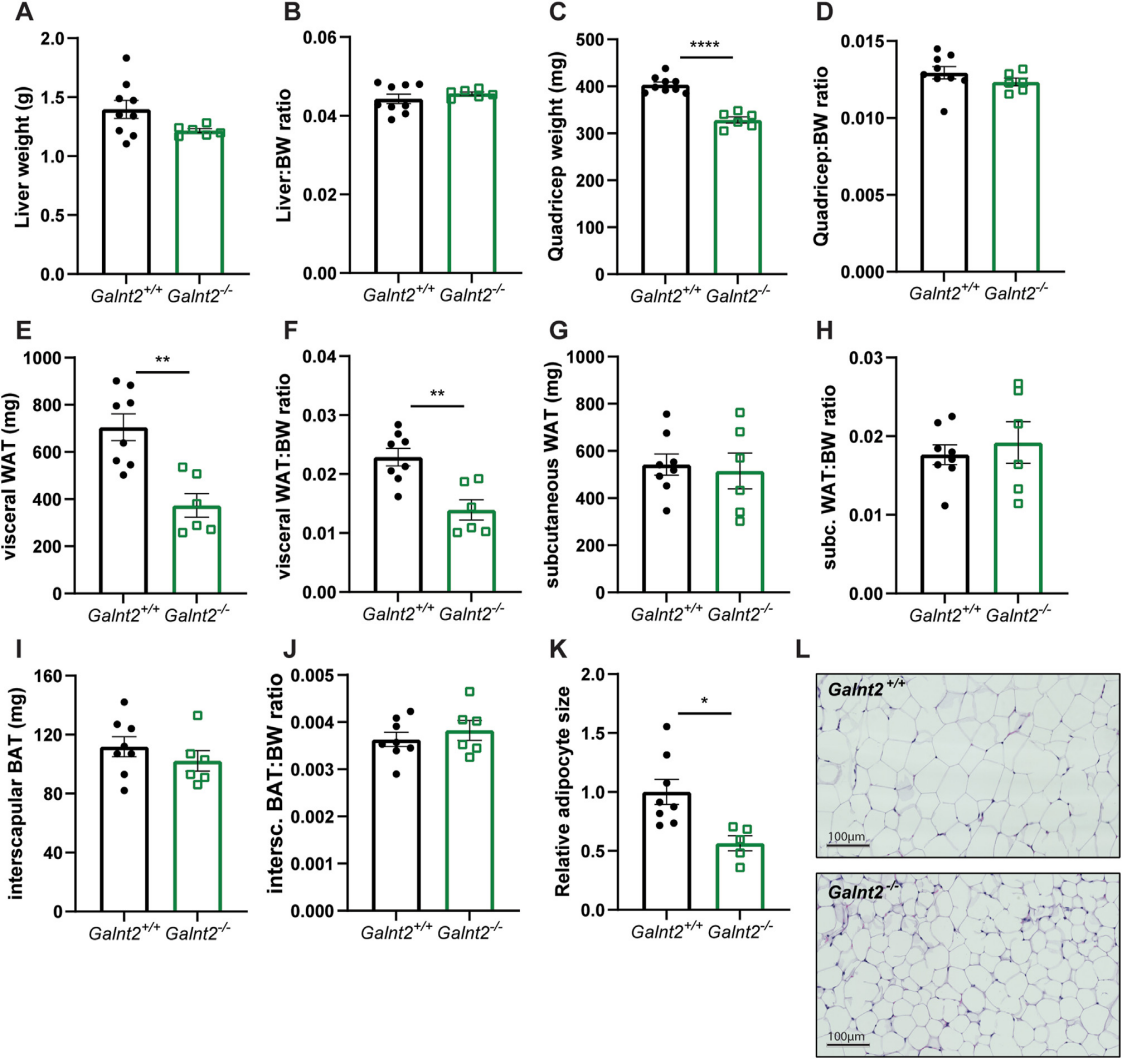
***Galnt2***^***−/−***^**mice display reduced quadriceps and visceral WAT weight and visceral WAT adipocyte size.** Metabolic organs were dissected from mice on a control diet after fasting for 4 h. (**A**) Liver weight, (**B**) liver-to-body weight ratio, (**C**) Quadriceps weight, (**D**) Quadricep-to-body weight ratio, (**E**) visceral WAT weight, (**F**) visceral WAT-to-body weight ratio, (**G**) subcutaneous WAT and (**H**) subcutaneous WAT-to-body weight ratio, (**I**) interscapular BAT and (**J**) interscapular BAT-to-body weight ratio is shown (n = 6–9, similar results obtained among three cohorts). (**K**) Adipocyte size quantification shown as relative adipocyte size (n = 5–8, size measures based on pixels). (**L**) H&E staining of visceral WAT slides from wild-type and *Galnt2*^*−/−*^ mice (representative images from n = 5–8). Data are presented as mean values ± SEM with ∗p < 0.05, ∗∗p < 0.01, ∗∗∗∗p < 0.0001.

**Supplementary Figure 4:**
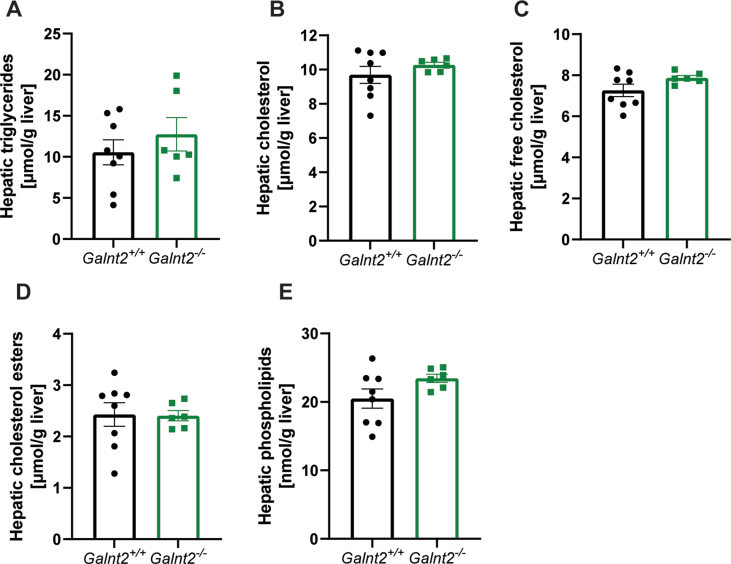
**No differences in hepatic lipid content in Galnt2**^**−/−**^**mice and wild-type littermates.** Hepatic (**A**) triglycerides, (**B**) total cholesterol, (**C**) free cholesterol, (**D**) cholesterol esters, and (**E**) phospholipids in control and *Galnt2*^*−/−*^ mice fed a control diet (n = 6–8). Data are presented as mean values ±SEM

**Supplementary Figure 5:**
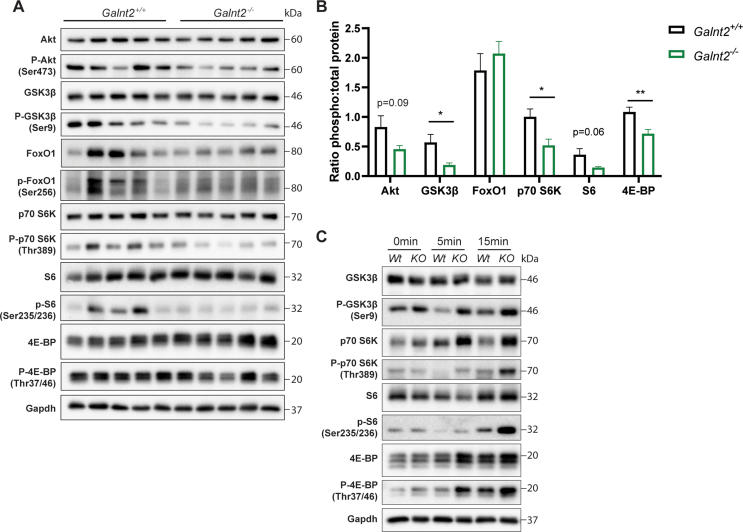
**The Akt/mTORC1 signaling pathway in unstimulated and insulin stimulated visceral WAT derived from *Galnt2***^***−/−***^**mice and wild-type littermates.** (**A**) Immunoblotting of total and phosphorylated Akt, Gsk3β, FoxO1, p70 S6K, S6, 4E-BP and housekeeping protein Gapdh in unstimulated visceral WAT harvested from control diet fed *Galnt2*^*−/−*^ mice and wild-type littermates (n = 5). (**B**) Quantification of immunoblot results shown by the ratio between phosphorylated versus total protein. (**C**) Immunoblotting of total and phosphorylated Gsk3β, p70 S6K, S6, 4E-BP and housekeeping protein Gapdh in visceral WAT stimulated with insulin for the indicated time points. Visceral WAT was derived from control diet fed *Galnt2*^*−/−*^ mice and wild-type littermates. Results shown are representative of two independent experiments. Data are presented as mean values ±SEM with ∗p < 0.05, ∗∗p < 0.01

To delineate the causes of reduced body weight gain in *Galnt2*^*−/−*^ mice and found a trend toward reduced liver weight (p = 0.08) but no differences in liver-to-body weight ratio when compared with wild-type littermates (Figure 3A,B). Also, no differences were observed in hepatic levels of triglycerides, free cholesterol, cholesterol esters and phospholipids between genotypes (Fig. S4). In line with reduced lean and fat mass (Figure 2C,F), *Galnt2*^*−/−*^ mice showed significant decreases in quadriceps and visceral white adipose tissue (WAT) weight. However, only the change in visceral WAT weight remained significant after normalization to body weight (Figure 3C–F). No differences between genotypes were observed in both the interscapular brown adipose tissue (BAT) and subcutaneous WAT weight (Figure 3G–J). The reduction in visceral WAT in *Galnt2*^*−/−*^ mice was associated with an almost two-fold decrease in visceral adipocyte size compared with wild-type littermates (Figure 3K,L). Akt/mTORC1 signaling is important for normal adipose tissue growth and adipogenesis [28], so we tested whether the Akt/mTORC1 signaling pathway was affected in visceral WAT of *Galnt2*^*−/−*^ mice. Upon immunoblotting of Akt and mTORC1 substrates, we observed a decrease in phosphorylated glycogen synthase kinase 3 beta (Gsk3β), ribosomal protein S6 kinase (p70 S6 kinase), ribosomal protein S6 and eukaryotic translation initiation factor 4E-binding protein 1 (4E-BP) (Figs. S5A and B). These findings suggest reduced Akt/mTORC1 signaling in absence of *Galnt2* in visceral WAT.

Taken together, murine *Galnt2* deficiency leads to changes in visceral WAT mass, adipocyte size and Akt signaling, and this extends the *in vitro* findings by Marucci and Di Paola et al., who suggested that GalNAc-T2 plays a role in adipocyte insulin signaling, maturation and adipogenesis [9,10]. Overall, these findings suggest a novel role for GALNT2 in visceral WAT.

### 3.5 Galnt2 deficiency does not alter insulin and glucose tolerance but affects the insulin receptor and insulin signaling

**Figure 4:**
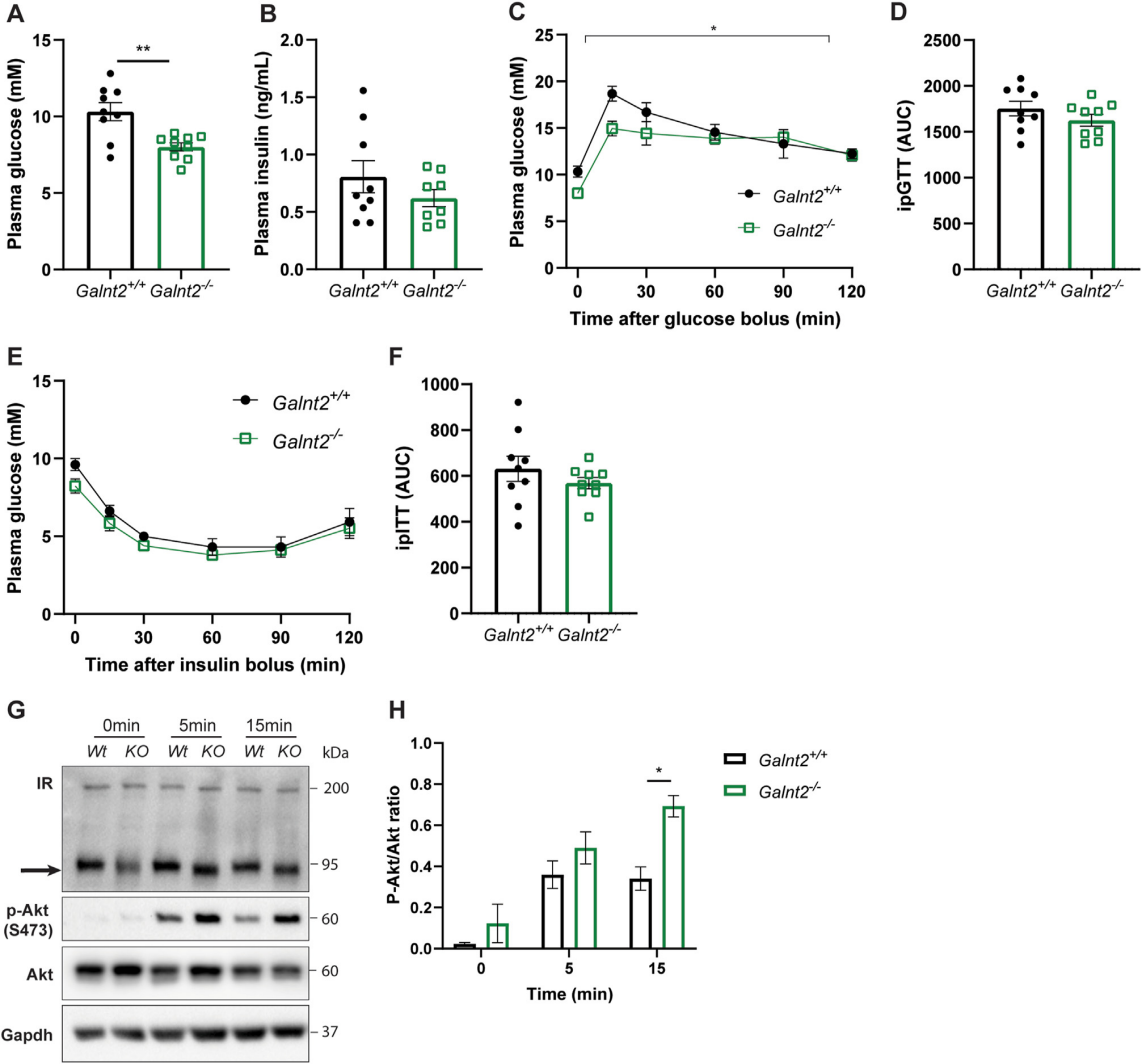
**Glucose tolerance and insulin signaling in *Galnt2***^***−/−***^**mice.** (**A**) Fasting plasma glucose and (**B**) insulin levels (n = 8–9). Wild-type and *Galnt2*^*−/−*^ mice on a control diet were fasted for 6 h followed by an intraperitoneal administration of glucose (2  g/kg of body weight) to assess (C) glucose tolerance and (D) the corresponding AUC (n = 9). Wild-type and Galnt2−/− mice were fasted for 6 h followed by an intraperitoneal administration of insulin (0.75 U/kg of body weight) to assess (**E**) insulin tolerance and (**F**) the corresponding AUC (n = 9). (**G**) Insulin signaling in visceral WAT was assessed by immunoblotting the insulin receptor (IR) with the arrow indicating a shift in molecular weight, P-Akt S473 and Akt after insulin stimulation for 0, 5 and 15 min and (**H**) quantified (n = 3). Data are presented as mean values ± SEM with ∗p < 0.05, ∗∗p < 0.01.

The combined effects on body weight gain and composition as well as visceral WAT prompted us to study potential effects of *Galnt2* deficiency on glucose homeostasis. While fasting plasma glucose levels were reduced in *Galnt2*^*−/−*^ mice, insulin levels were not affected (Figure 4A,B). Despite being significantly smaller, no pronounced differences in glucose or insulin tolerance were observed in *Galnt2*^*−/−*^ mice compared with wild-type littermates (Figure 4C–F). Next, we assessed the *ex vivo* insulin signaling in visceral WAT as changes in insulin signaling have been shown to alter adipocyte lipid storage and breakdown [29]. We found a rapid and enhanced *Akt* phosphorylation upon 5 and 15 min of insulin treatment in visceral WAT depots of *Galnt2*^*−/−*^ mice (Figure 4G,H) compared with controls. Downstream levels of phosphorylated Gsk3β, p70 S6 kinase, S6 and 4E-BP were also increased after 15 min of insulin stimulation (Fig. S5C), suggesting more sensitive activation of the Akt/mTORC1 pathway in visceral WAT of *Galnt2*^*−/−*^ mice compared with wild-type littermates. These findings suggest that loss of *Galnt2* leads to changes in response to insulin, resulting in aberrant rapid insulin receptor activation followed by increased Akt signaling.

Interestingly, when immunoblotting for the insulin receptor, we observed that the apparent molecular weight of the mature insulin receptor was reduced in visceral WAT of *Galnt2*^*−/−*^ mice (Figure 4G, arrow) compared with controls, suggesting that it may be a target of GalNAc-T2. Collectively, these data indicate that *Galnt2* deficiency has only mild effects on glucose and insulin tolerance. However, loss of Galnt2 results in altered insulin signaling and a change in the molecular weight of the insulin receptor, suggesting that the insulin receptor may be a direct target of GalNAc-T2.

### 3.6 The insulin receptor is a new target of GalNAc-T2 mediated O-glycosylation

**Figure 5:**
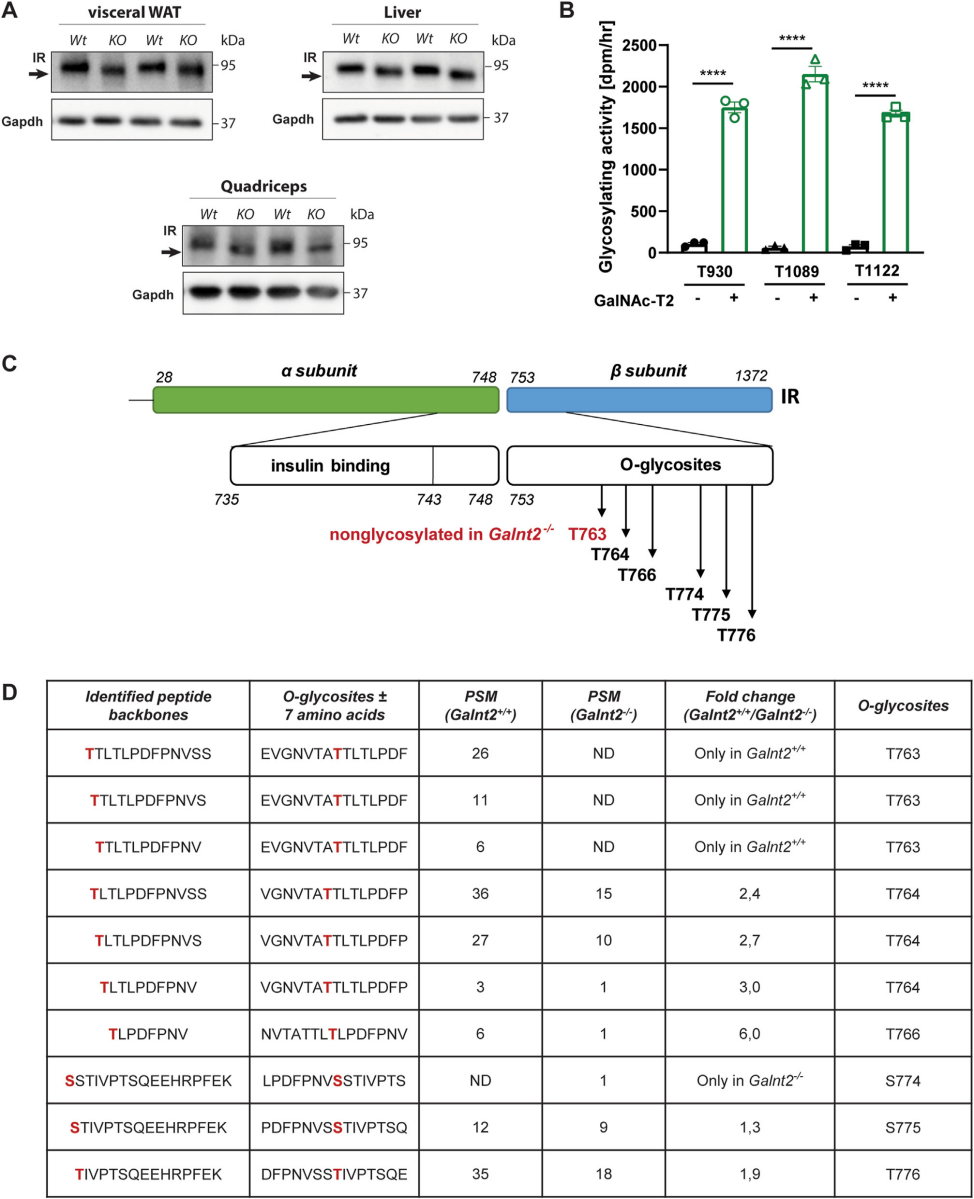
**The insulin receptor is novel target of GalNAc-T2 mediated O-glycosylation**. (**A**) The insulin receptor (IR) was immunoblotted in visceral WAT, liver and quadriceps (shown n = 2 animals per genotype, but blot was repeated for each mouse in this study). (**B**) Activity of GalNAc-T2 as rate of transfer using synthetic IR peptide substrates containing potential glycosylation sites identified using *in silico* analysis: T930, T1089 and T1122 (n = 3). The background activity was subtracted, and data are presented as mean values ± SEM with ∗p < 0.05, ∗∗p < 0.01. (**C**) Schematic representation of the insulin receptor containing the identified O-glycosites *in vivo*. (**D**) Table with site-specific identification of O-glycosites of the insulin receptor in livers from wild-type and *Galnt2*^*−/−*^ mice using the EXoO method. PSM = peptide-to-spectrum matches, ND = not detectable.

The shift in the apparent molecular weight of the mature insulin receptor was identified in visceral WAT, liver and quadriceps derived from *Galnt2*^*−/−*^ mice (Figure 5A). This pointed toward a difference in the glycosylation state of the insulin receptor between genotypes in different metabolic organs. To study this, we performed *in silico* analysis using the ISOGlyP (Isoform Specific O-Glycosylation Prediction, http://isoglyp.utep.edu/index.php) server. This revealed three potential O-linked glycosylation sites in the insulin receptor, at T930, T1089 and T1122. O-glycosylation assays using synthetic peptide substrates suggest that GalNAc-T2 modifies the three putative sites *in vitro* (Figure 5B). Next, we used a mass spectrometry based-method to map site-specific O-GalNAcylation sites, called extraction of O-linked glycopeptides (EXoO), using livers derived from wild-type and *Galnt2*^*−/−*^ mice [19]. A total of six O-glycosites were detected at the insulin receptor. These included T763, T764, T766, S774, S775 and T776 that are part of an extracellular domain (amino acids 753–946) and in close proximity to the insulin-binding domain (amino acids 735–743) (Schematically represented in Figure 5C). Differential analysis revealed that T763 was detected in livers derived from wild-type mice with a total peptide-to-spectrum matches (PSM) of 43, but it was not detected in livers derived from *Galnt2*^*−/−*^ mice (Figure 5D). The PSM number of other O-glycosylated sites was significantly decreased in liver samples derived from *Galnt2*^*−/−*^ mice ranging from 1.3 to 6-fold decrease (Figure 5D). There was an exception for S774, which was only detected in the liver samples derived from *Galnt2*^*−/−*^ mice, but the low PSM number suggests a low abundance. In conclusion, the insulin receptor harbored decreased levels of O-glycosylation and an absence of O-glycosylation at T763 in livers of *Galnt2*^*−/−*^ mice, supporting the hypothesis that the insulin receptor is a novel target of GalNAc-T2.

### 3.7 *Galnt2*^*−/−*^ mice exhibit increased plasma NEFA levels but normal adipose tissue lipolysis

**Figure 6:**
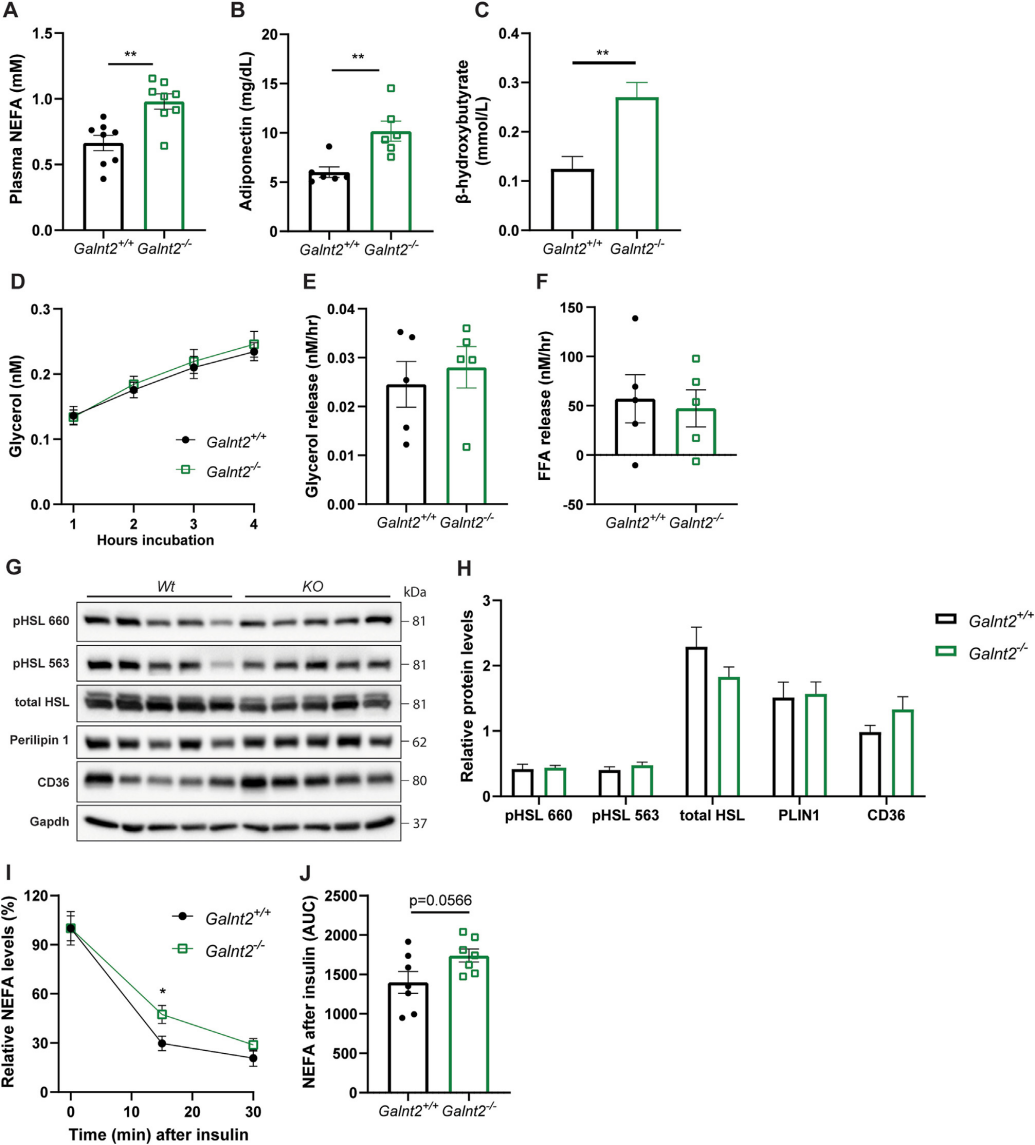
**Assessment of visceral WAT lipolysis in *Galnt2***^***−/−***^**mice**. (**A**) Fasting plasma NEFA levels (n = 8, repeated throughout 4 cohorts of n = 6–10), (**B**) adiponectin levels (n = 6) and (**C**) β-hydroxybutyrate (n = 10–12). (**D**) *Ex vivo* visceral WAT basal lipolysis as measured by glycerol release, (**E**) hourly glycerol release and (**F**) free fatty acid release (n = 5). (**G**) Western blotting of lipolytic markers and (**H**) quantification of the western blots (n = 5). Protein levels are normalized against Gapdh and pHSL 660 and pHSL 563 are relative compared to the total HSL. (**I**) Plasma non-esterified fatty acid levels after 0.75U/kg insulin injection and (**J**) the corresponding AUC (n = 7). Experiments were performed using mice that were on control diet. Data are presented as mean values ± SEM with ∗p < 0.05, ∗∗p < 0.01.

**Supplementary Figure 6:**
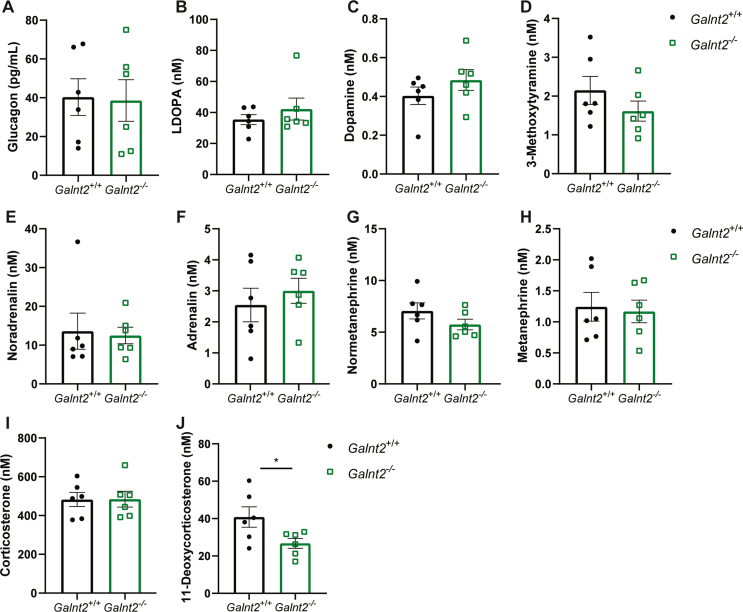
**Sympathetic outflow is not affected in *Galnt2***^***−/−***^**mice**. Measurement of (**A**) glucagon, (**B**) l-DOPA, (**C**) dopamine, (**D**) 3-methoxytyramine, (**E**) noradrenalin, (**F**) adrenalin, (**G**) normetanephrine, (**H**) metanephrine, (**I**) corticosterone, (**J**) 11-deoxycorticosterone levels in plasma. Data presented as mean values ±SEM with ∗p < 0.05 (n = 6 per genotype)

To further explore the mechanism underlying reduced visceral WAT mass, we assessed whether *Galnt2* deficiency affects adipocyte function. Interestingly, we found markedly increased fasting plasma non-esterified fatty acid (NEFA) levels in *Galnt2*^*−/−*^ mice (Figure 6A); this may be due to increased adipocyte lipolysis. Fasting plasma adiponectin levels, an adipocyte-derived adipokine involved in glucose metabolism and fatty acid oxidation, were increased almost two-fold (Figure 6B). In line with increased NEFA levels, we observed a more than two-fold increase in β-hydroxybutyrate, one of the main ketone bodies (Figure 6C). Intrinsic *ex vivo* lipolysis of visceral WAT pads as measured by the release of glycerol (Figure 6D,E) and free fatty acids (Figure 6F), however, was not affected in *Galnt2*^*−/−*^ mice. Accordingly, no differences were observed in proteins involved in the adipose tissue lipolysis pathway, including phosphorylated and total hormone-sensitive lipase, perilipin 1 and CD36 (Figure 6G,H). In addition, we did not observe a strong impairment in insulin-mediated suppression of lipolysis, with *Galnt2*^*−/−*^ mice only showing a mildly delayed insulin-mediated turnover of free fatty acids compared with wild-type littermates (Figure 6I,J). As adipose tissue lipolysis is also under the control of sympathetic activity, we also tested whether there were changes in plasma levels of lipolytic hormones, including glucagon, catecholamines, corticosterone and intermediate products. We found no significant differences in these hormones apart from a slight but significant decrease in 11-deoxycorticosterone in *Galnt2*^*−/−*^ mice compared with controls (Fig. S6). Taken together, our studies do not support abnormal adipose tissue lipolysis in *Galnt2*^*−/−*^ mice but suggest altered peripheral NEFA uptake or metabolism.

### 3.8 *Galnt2* deficient mice rely more on lipids as a substrate for energy

**Figure 7:**
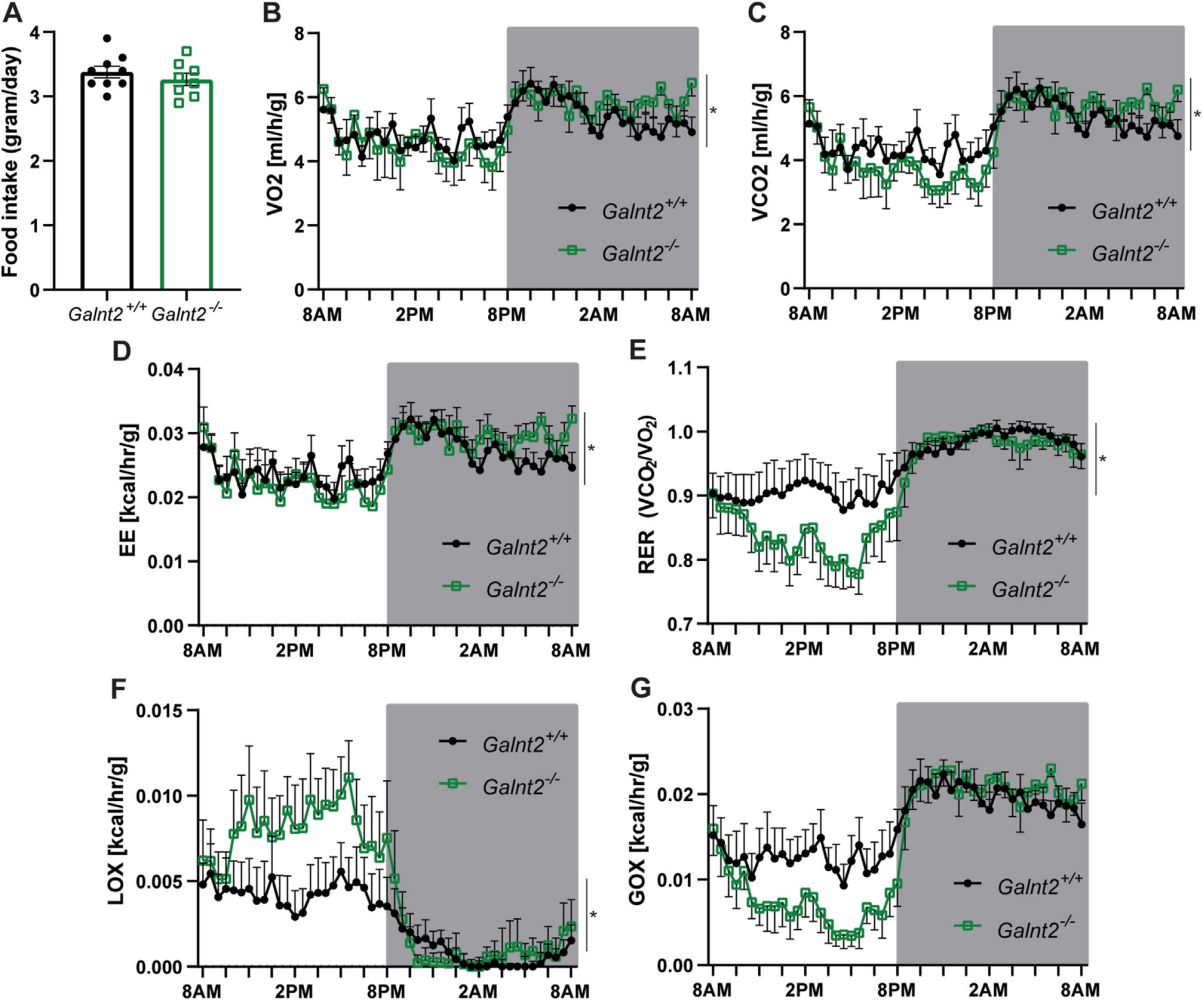
**Global energy homeostasis is affected in *Galnt2***^***−/−***^**mice**. Wild-type and *Galnt2*^*−/−*^ mice were subjected to an indirect calorimetric study with a 12-hour light/dark cycle on a control diet. (**A**) Food intake of wild-type and *Galnt2*^*−/−*^ mice. (**B**) Oxygen consumption and (**C**) carbon dioxide production were measured and (**D**) energy expenditure, (**E**) respiratory exchange ratio, (**F**) lipid oxidation and (**G**) glucose oxidation were calculated (n = 7–8). Generalized linear model (GLM) statistics were used for B-D, and 2-way ANOVA was used for E-G using *CalR* [13]. The indirect calorimetry experiments were repeated 3 times among different cohorts, and the results were similar. Data are corrected for lean mass and presented as mean values ± SEM with ∗p < 0.05.

**Supplementary Figure 7:**
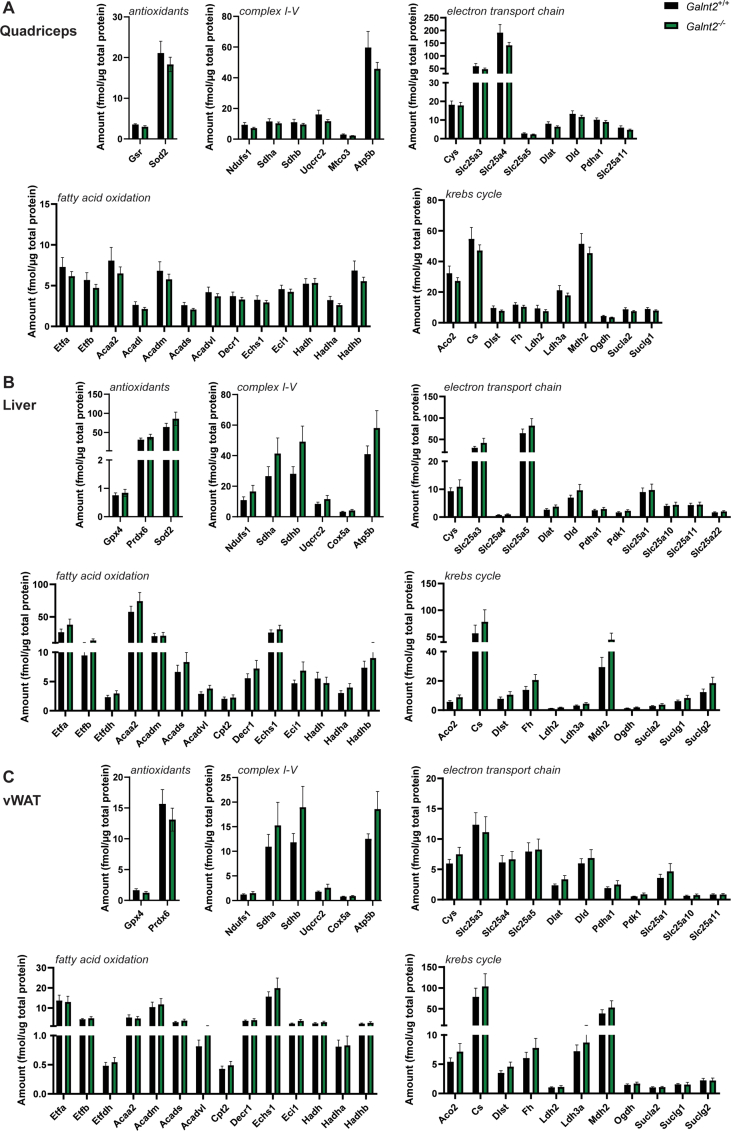
**Proteins involved in mitochondrial respiration in quadriceps, liver and visceral WAT are not affected in *Galnt2***^***−/−***^**mice**. Targeted proteomics using qConCATamers targeting different proteins involved in mitochondrial respiration in (**A**) quadriceps, (**B**) liver and (**C**) visceral WAT protein lysates derived from *Galnt2*^*−/−*^ mice and wild-type littermates (n = 5–6). Data presented as mean values ±SEM

*Galnt2*^*−/−*^ mice display reduced visceral WAT mass and adipocyte size, while plasma NEFA, adiponectin and β-hydroxybutyrate levels are increased without apparent changes in intrinsic adipocyte lipolysis. To gain more insight into the mechanisms underlying this metabolic phenotype, we examined the energy metabolism of *Galnt2*^*−/−*^ mice using indirect calorimetry. Food intake was similar between *Galnt2*^*−/−*^ mice and wild-type littermates (Figure 7A). Interestingly, we found differences in oxygen consumption, carbon dioxide production, energy expenditure and respiratory exchange ratio (RER) between genotypes (Figure 7B–E). Compared with wild-type littermates, *Galnt2*^*−/−*^ mice showed a marked decrease in RER in the inactive (light) phase, indicating increased preference for lipids as a fuel source. Indeed, when calculating the oxidative substrate preference, we found a profound increase in lipid oxidation (Figure 7F) and a decrease in glucose oxidation (Figure 7G) in *Galnt2*^*−/−*^ mice during the inactive phase. These differences, however, were not observed in the active (dark) phase and were not related to differences in protein levels of mitochondrial respiration markers in quadriceps, liver or visceral WAT (Fig. S7). Our studies reveal a surprising novel role for GalNAc-T2 in the use of lipids as oxidative fuel in the inactive phase.

### 3.9 *Galnt2* and its protein substrates

**Figure 8:**
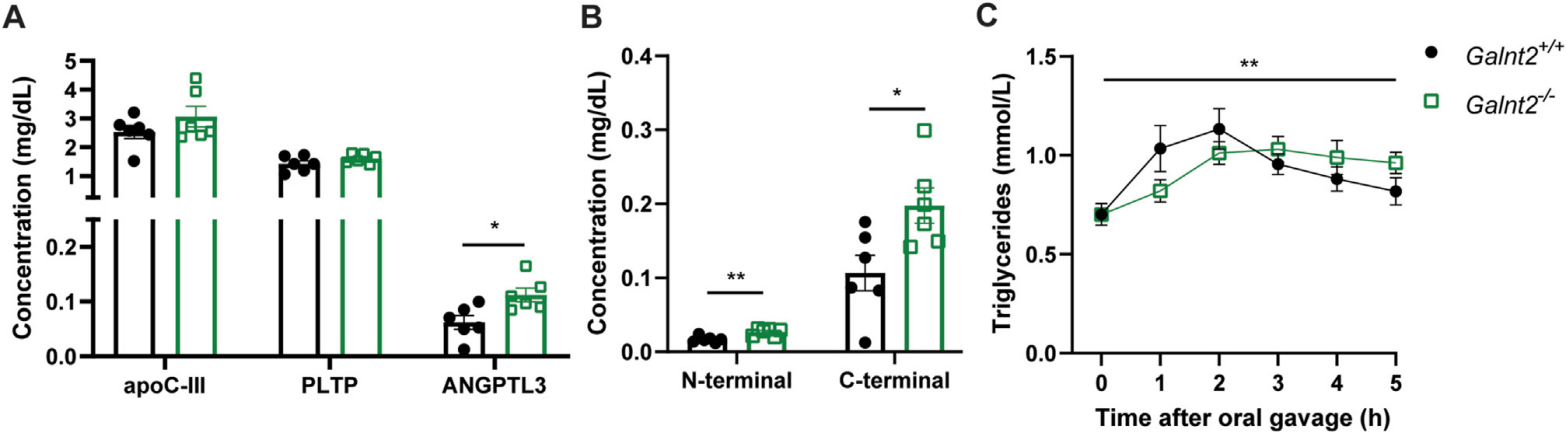
**Substrates of GALNT2**. (**A**) Targeted proteomics of apoC-III, PLTP and ANGPTL3 in plasma regulating lipid metabolism (n = 6). (**B**) Targeted proteomics of ANGPTL3 with peptides targeting the N-terminal and C-terminal site. (**C**) Oral fat tolerance test represented as triglyceride levels at 0, 1, 2, 3, 4 and 5 h after olive oil bolus (n = 9). Data presented as mean values ± SEM with ∗p < 0.05, ∗∗p < 0.01.

Finally, we focused on substrates of *Galnt2* that have thus far been described in the literature, i.e., *apoC-III* and *ANGPTL3* (both inhibitors of lipoprotein lipase (LPL), which normally mediates the lipolysis of triglyceride-rich lipoproteins) and *PLTP* [[3], [4], [5]]. To assess potential effects of *Galnt2* deficiency on protein concentrations, we used a targeted proteomics approach in which all substrates were quantified simultaneously in plasma samples. This analysis did not reveal significant differences in the concentrations of apoC-III and PLTP (Figure 8A). The earlier proposed altered glycosylation of these proteins, therefore, did not appear to have resulted in quantitative differences in plasma between genotypes in our study. We did observe increased levels of ANGPTL3 in plasma of *Galnt2*^*−/−*^ mice (Figure 8A). ANGPTL3 is known to be cleaved at the proprotein convertase processing site, resulting in a separate N-terminal and C-terminal site, and it is suggested that the N-terminal site contains the LPL binding motif to inhibit LPL in the circulation [30]. Previous studies suggested that GalNAc-T2 is an important regulator in ANGPTL3 cleavage [4,31]. We observed a significant increase in both the N- and C-terminal site of ANGPTL3 in plasma and a higher concentration of the C-terminal site compared with the N-terminal site (Figure 8B). The detected fragments can be part of the complete protein or the two separate fragments. These results cannot rule out qualitative effects due to loss of GalNAc-T2 mediated O-linked glycosylation of these substrates or subsequent downstream effects such as lipolysis. To assess the actual physiological consequences of all above findings, we measured postprandial triglyceride levels following an oral fat tolerance test. *Galnt2*^*−/−*^ mice display delayed triglyceride absorption as well as delayed clearance compared to controls (Figure 8C), suggesting alterations in the postprandial triglyceride response. Although the current study also reveals that the insulin receptor is yet another substrate for GalNAc-T2, the findings combined highlight the complexity of the metabolic phenotype of a complete loss of GalNAc-T2 in mice.

## 4 Discussion

We showed that GALNT2 plays an important dynamic role in whole-body energy homeostasis. First, we revealed significant associations between *GALNT2* gene variation, BMI and fat mass in humans. Our studies in *Galnt2*^*−/−*^ mice subsequently reveal decreased adiposity, altered insulin signaling and a shift in energy substrate utilization during the inactive phase. GalNAc-T2 has previously been shown to exert systemic effects on plasma lipids through post-translational modification, i.e., O-linked glycosylation, of proteins that are secreted by the liver [[3], [4], [5]]. The current study provides the first evidence that GalNAc-T2 can also have local effects through O-glycosylation of the insulin receptor.

We performed genetic associations with biological traits using a common variant of *GALNT2*, namely rs4846914, extracted from UK Biobank GWAS data. The A allele was significantly associated with increased HDL cholesterol levels and decreased triglyceride levels and overall reduced adiposity (traits like body fat percentage, whole-body fat mass and body mass index were decreased). In contrast, carriers of rare loss of function (LOF) variants were previously shown to have both decreased plasma HDL cholesterol and triglyceride levels [5]. Furthermore, GALNT2-CDG patients not only exhibit reduced levels of plasma HDL cholesterol and triglycerides, but also dysmorphic facial features, short stature and developmental delay [6]. The latter clinical findings are recapitulated in various *Galnt2*-deficient animal models, and those models also show decreased adiposity. There is a discrepancy in the directionality between common SNPs in GWAS studies, rare LOF variants and knock-out animal models. Thus, common variants result in milder phenotypes as compared with the severe pathophysiological phenotypes observed in carriers of rare LOF variants.

We observed reduced body weight in three to nine-week-old male and female *Galnt2*^*−/−*^ mice and decreased body length and body weight gain in adult male *Galnt2*^*−/−*^ mice upon control or HFD feeding. This observation is in line with previous studies showing reduced body weight and decreased stature in human carriers of *GALNT2* variants, male *Galnt2*^*−/−*^ mice and cattle carrying *Galnt2* variants [6,7]. We demonstrate that the reduction in body weight is the result of a decrease in both lean and fat mass; this was exacerbated when *Galnt2*^*−/−*^ mice were fed a HFD. However, when adjusted for body weight, visceral WAT was the only tissue that was significantly reduced in *Galnt2*^*−/−*^ mice, and this abnormality coincided with reduced visceral WAT adipocyte size. In line, previous studies in pre-adipocytes also showed that GalNAc-T2 overexpression improved insulin signaling, stimulated adipocyte maturation and led to enlarged mature adipocytes [9,10]. The absence of changes in subcutaneous WAT in our study can be explained by differences in plasticity, metabolic function and immune cell composition between visceral and subcutaneous WAT [[32], [33], [34]]. For instance, visceral WAT is considered to exert more adverse effects on health, is strongly associated with insulin resistance and represents the primary fat storage depot during the initial phase of obesity [32,33]. One possible explanation for the observed differences is that GalNAc-T2 exerts local actions via downstream effects of its substrates in visceral but not subcutaneous WAT. For example, it has been shown that ANGPTL3, one of the proposed GalNAc-T2 substrates, plays an important role in the uptake of fat into visceral WAT, but this is not described for subcutaneous WAT [35]. Adipose tissue is an important regulator of insulin sensitivity and glucose tolerance, and smaller adipocytes are often linked to improved insulin sensitivity and glucose tolerance [34]. Standard glucose and insulin tolerance tests, however, did not reveal notable changes in these parameters. We did observe significant reductions in plasma glucose levels: this could be due to decreased gluconeogenesis, reduced hepatic glucose production or glucose transport issues.

**Supplementary Figure 8:**
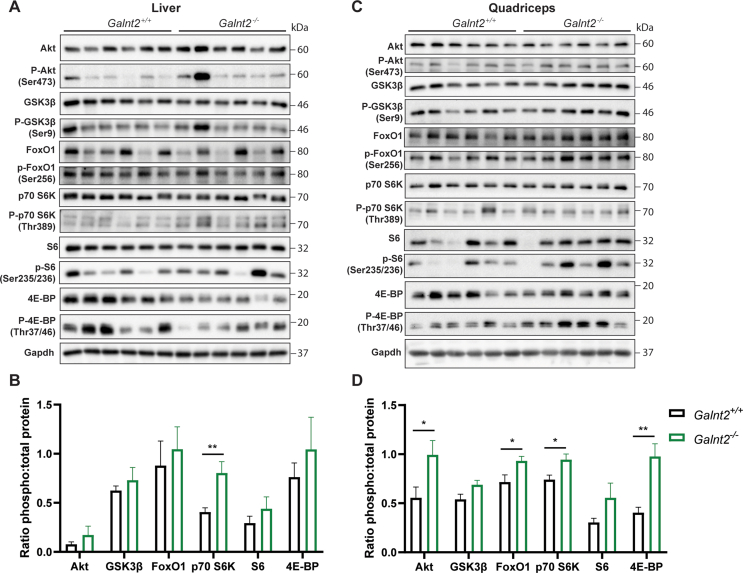
**The Akt/mTORC1 signaling pathway in liver and quadriceps derived from *Galnt2***^***−/−***^**mice and wild-type littermates.** (**A**) Immunoblotting of total and phosphorylated Akt, Gsk3β, FoxO1, p70 S6K, S6, 4E-BP and housekeeping protein Gapdh in livers, (**B**) ratio phosphorylated protein versus total protein. (**C**) Immunoblotting of the above mentioned markers in quadriceps, (**D**) ratio phosphorylated protein versus total protein. Tissues were harvested from control diet fed *Galnt2*^*−/−*^ mice and wild-type littermates (n = 6). Data are presented as mean values ±SEM with ∗p < 0.05, ∗∗p < 0.01

The smaller adipocytes, reduced visceral WAT and body weight of *Galnt2*^*−/−*^ mice resemble whole-body and adipocyte-specific *S6k1*^*−/−*^ mice and other models in which mTORC1 signaling is disrupted [[36], [37], [38]]. In visceral WAT harvested from fasted mice on a control diet, we likewise found reduced phosphorylated protein levels of Akt and downstream targets of Akt and mTORC1 (Gsk3β, Foxo1, S6K1, S6, and 4E-BP) in visceral WAT; this lends support for the involvement of changes in Akt/mTORC1 signaling in explaining smaller adipocytes, visceral WAT and body size (Figs. S5A and B). Interestingly, *ex vivo* insulin stimulation of visceral WAT led to a transient increase in phosphorylated Akt in *Galnt2*^*−/−*^ mice compared with wild-type littermates (Figure 4G) and was accompanied by an increase in downstream Akt and mTORC1 signaling (Fig. S5C). This suggests increased activity of the insulin receptor caused by the loss of O-glycosylation. Studying Akt and mTORC1 signaling in the liver of *Galnt2*^*−/−*^ mice compared with wild-type littermates only revealed increased phosphorylation of p70 S6K with no further increase of S6 activation (Figs. S8A and B). In quadriceps, we observed an overall increase in Akt/mTORC1 signaling (Figs. S8C and D), which was not further studied. However, as GalNAc-T2 is expressed only at low levels in muscle (data not shown), it remains unclear whether this is a direct or indirect effect. Taken together, our *Galnt2* mouse studies reveal that GalNAc-T2 plays a new physiological role in adipocyte-specific insulin signaling in visceral WAT.

Our *in vitro* studies show that the insulin receptor can be O-glycosylated by GalNAc-T2 at three threonine residues. Although the insulin receptor has been reported to be heavily glycosylated with both N- and O-glycans, the key enzymes responsible for mediating the glycosylation process are currently unknown. Though it is generally assumed that N-linked glycosylation is essential for insulin binding and autophosphorylation [[39], [40], [41]], a role for GalNAc-T enzymes in O-linked glycosylation of the insulin receptor has, to our knowledge, not been described. Using a recently developed EXoO method [19], we show that the insulin receptor lacks O-glycosylation at T763 accompanied by reduced O-glycosylation at T764, T766, S775 and T776 in *Galnt2*^*−/−*^ mice compared with wild-type littermates. To our knowledge, we are the first to show that the insulin receptor is a substrate of GalNAc-T2. The respective threonine and serine residues are in close proximity to an insulin-binding domain, and alterations in O-linked glycosylation at these sites may impact the insulin receptor activation by changes in insulin binding and subsequent Akt signaling.

*Galnt2*^*−/−*^ mice presented with increased plasma NEFA and adiponectin levels. In line, we observed increased plasma β-hydroxybutyrate levels as a measure of ketone bodies (Figure 6C), in *Galnt2*^*−/−*^ compared with wild-type mice [42]. Although augmented ketone bodies are indicative for increased mitochondrial oxidation in the livers of *Galnt2*^*−/−*^ mice, no changes were seen in mitochondrial proteins in the liver (Fig. S7). However, we cannot rule out changes of protein levels at other time points, such as after prolonged fasting, throughout the light/dark cycle or related to changes in the activity of mitochondrial proteins, e.g., by posttranslational modifications. While these findings point at altered adipocyte triglyceride lipolysis and fatty acid oxidation [[43], [44], [45]], we did not observe abnormal intrinsic adipose tissue lipolysis. However, we identified profound changes in substrate utilization in indirect calorimetry studies. *Galnt2*^*−/−*^ mice predominantly use lipids for oxidation in the inactive phase but rapidly switch to glucose oxidation during the active phase as if they were wild-type animals. The increased use of lipids as substrates during the fasting/resting period is consistent with the decreased fasting plasma glucose levels in *Galnt2*^*−/−*^ mice, meaning that less glucose is available to be used as substrate. These findings point toward a problem in the production, uptake or utilization of glucose during fasting and a problem with NEFA metabolism or uptake by adipose tissue. A swift transition in the use of substrates between fasting and refeeding is usually attenuated in pathophysiological models [46]. Increased lipid oxidation may be due to the activation of NEFA mobilization from white adipose tissue and fatty acid transport into mitochondria for oxidation in energy-demanding tissues. Mice are normally fasting in the light phase, which lowers insulin levels, leading to enhanced adipose tissue triglyceride hydrolysis and delivery of NEFA [46]. This leads us to speculate that this effect could be related to impaired glucose production or to our finding that *Galnt2*^*−/−*^ mice display altered adipocyte-specific insulin action. Our observations suggest enhanced metabolic flexibility with regard to substrate use, which is in line with the observed elevated plasma adiponectin levels in *Galnt2*^*−/−*^ mice [46,47].

GalNAc transferases all catalyze the same enzymatic reaction but exert distinct effects due to differential tissue expression, enzyme activity and substrate specificity [48]. When considering the actual potential mediators of the various observations in our mouse model, i.e., the substrates of GalNAc-T2, the studies published thus far have focused on the association between *GALNT2* and plasma HDL cholesterol levels. This has rendered apoC-III and Angptl3 as GalNAc-T2 known substrates [[3], [4], [5]], but this will only be a fraction of its total number of targets. To explain the plasma lipid phenotype, Khetarpal et al. also identified PLTP as a third substrate mediating the HDL cholesterol phenotype [5]. The post-translational modifications of ApoC-III, ANGPTL3 and PLTP by GalNac-T2 can affect protein concentrations, for example, through changing further protein processing, synthesis or catabolism. In the above studies, antibodies were used to detect proteins of interest, which showed that GALNT2 affects isoforms of apoC-III and cleavage of the N-terminal part of Angptl3 [[3], [4], [5],^31^]. To address effects in a quantitative manner in the current study, we used targeted proteomics in which unique peptides labeled with stable isotopes are used to quantify the concentrations of peptides of endogenous proteins in one single sample preparation [24,25]. Our *Galnt2*^*−/−*^ mice presented increased plasma ANGPTL3 protein levels. Several studies have reported a role for GalNac-T2 in regulating ANGPTL3 cleavage through O-linked glycosylation of a threonine amino acid adjacent to a proprotein convertase processing site, thereby blocking cleavage *in vitro* and *in vivo* [4,31]. Actual cleavage yields an N-terminal domain containing the LPL binding motif and a C-terminal domain. Mice overexpressing the N-terminal fraction of ANGPTL3 indeed exhibit an increased plasma LPL inhibitory capacity and increased plasma triglyceride levels, however, a cleavage-resistant mutant of ANGPTL3 is still able to inhibit LPL, suggesting that the unprocessed protein is also able to inhibit LPL [30]. Additionally, inhibiting or promoting ANGPTL3 cleavage, by Galnt2 overexpression or suppression, did not alter plasma triglyceride levels in mice, again suggesting that ANGPTL3 cleavage is not essential for its LPL inhibitory capacity [31]. In our mass spectrometry measurements, we used one peptide that detects the N-terminal part, upstream of a proprotein convertase cleavage site, and one peptide that detects the C-terminal part of ANGPTL3 and both were increased in *Galnt2*^*−/−*^ mice. With the previous studies in mind, an increase in ANGPTL3 (full protein or cleaved fragments) in *Galnt2*^*−/−*^ mice is expected to increase inhibition of LPL and reduce hydrolysis of triglyceride-rich lipoproteins. However, in the fasting state as well as after an oral fat load, *Galnt2*^*−/−*^ mice do not present with marked changes in plasma triglycerides. Apparently, the role of GalNac-T2 in regulating triglycerides via ANGPTL3 in our mice is limited. This may be due to quantitative changes in other GalNac-T2 substrates, which brings us to apoC-III and PLTP concentrations that were not found to be different between *Galnt2*^*−/−*^ mice and controls. Attenuated O-linked glycosylation may, however, change the biochemical and biological properties of proteins that are not reflected by changes in concentrations. For example, Holleboom et al. showed that in the post-prandial phase, hypoglycosylated apoC-III of individuals with a loss-of-function mutation in *GALNT2* is not residing on triglyceride-rich lipoproteins but on HDL [3]. Clearly, it is very challenging to pursue the downstream effects of attenuated GalNAc-T2 mediated O-linked glycosylation of multiple substrates that occur simultaneously, especially in a whole body knockout setting.

## 5 Conclusion

In humans, cattle and mice, loss of GALNT2 results in a syndrome characterized by an intriguing metabolic phenotype with decreased body weight and stature. In *Galnt2*^*−/−*^ mice, we observed a metabolic phenotype that combines decreased adiposity, changes in adipocyte function and altered energy substrate utilization. Our findings show that the insulin receptor is another GalNAc-T2 substrate that may be responsible for local rather than systemic effects. The local effects are of special interest when considering the tissue-specific role of GalNAc-T2 and other members of the GalNAc-T glycosyltransferase family that catalyze the same reaction. They may or may not be capable of compensating for the loss of GalNAc-T2 activity. Tissue-specific knockout mouse models are needed to further dissect the role of GalNAc-T2 and its substrates in various metabolic and non-metabolic tissues. The current study reveals that GalNAc-T2 has a previously unknown role in whole-body energy homeostasis: this may be due to glycosylation of the insulin receptor and thereby affect insulin signaling.
