# Supplementary material for: A novel role for GalNAc-T2 dependent glycosylation in energy homeostasis
Source: Mol Metab. 2022 Mar 15;60:101472. doi: 10.1016/j.molmet.2022.101472 (PMC9019398; doi:10.1016/j.molmet.2022.101472)
Supplement: Multimedia component 9 [file mmc1.docx]

**Supplementary Table 1. Significant genetic association of *GALNT2* SNP rs4846914 with**

**152 continuous traits and biomarkers using the UK Biobank.** Columns represent the phenotype, phenotype code, beta_meta as effect size of the alternative allele with se_meta as estimated standard error and pval_meta are the p-value of the beta_meta significance test.

| **Phenotype** | **Code** | **beta_meta** | **se_meta** | **pval_meta** |
| --- | --- | --- | --- | --- |
| HDL cholesterol | 30760 | 8.581e-02 | 2.566e-03 | 2.72E-245 |
| Apolipoprotein A | 30630 | 7.157e-02 | 2.530e-03 | 5.35E-176 |
| Triglycerides | 30870 | -6.376e-02 | 2.512e-03 | 4.06E-142 |
| Apolipoprotein B | 30640 | -2.729e-02 | 2.518e-03 | 2.31E-27 |
| Vitamin D | 30890 | 1.537e-02 | 2.443e-03 | 3.13E-10 |
| Aspartate aminotransferase | 30650 | -1.416e-02 | 2.426e-03 | 5.36E-09 |
| Lipoprotein A | 30790 | 1.382e-02 | 2.813e-03 | 8.92E-07 |
| Calcium | 30680 | 9.353e-03 | 2.552e-03 | 2.47E-04 |
| Cholesterol | 30690 | 8.799e-03 | 2.439e-03 | 3.10E-04 |
| Urate | 30880 | -7.695e-03 | 2.219e-03 | 5.24E-04 |
| C-reactive protein | 30710 | -8.592e-03 | 2.561e-03 | 7.93E-04 |
| SHBG | 30830 | 7.778e-03 | 2.435e-03 | 1.41E-03 |
| Cystatin C | 30720 | -7.773e-03 | 2.454e-03 | 1.54E-03 |
| LDL direct | 30780 | -5.799e-03 | 2.425e-03 | 1.68E-02 |
| Oestradiol | 30800 | 1.094e-02 | 4.661e-03 | 1.89E-02 |
| Urea | 30670 | -5.234e-03 | 2.358e-03 | 2.65E-02 |
| Total bilirubin | 30840 | -5.317e-03 | 2.586e-03 | 3.98E-02 |
| Phosphate | 30810 | 4.721e-03 | 2.506e-03 | 5.96E-02 |
| Alanine aminotransferase | 30620 | -3.584e-03 | 2.316e-03 | 1.22E-01 |
| Creatinine | 30700 | -2.499e-03 | 2.080e-03 | 2.30E-01 |
| Direct bilirubin | 30660 | 2.849e-03 | 2.717e-03 | 2.94E-01 |
| Rheumatoid factor | 30820 | -7.125e-03 | 7.565e-03 | 3.46E-01 |
| Total protein | 30860 | 2.422e-03 | 2.590e-03 | 3.50E-01 |
| Albumin | 30600 | -1.547e-03 | 2.540e-03 | 5.42E-01 |
| IGF-1 | 30770 | -1.476e-03 | 2.532e-03 | 5.60E-01 |
| Gamma glutamyltransferase | 30730 | -1.098e-03 | 2.424e-03 | 6.50E-01 |
| Testosterone | 30850 | 4.867e-04 | 1.393e-03 | 7.27E-01 |
| Glucose | 30740 | 7.833e-04 | 2.481e-03 | 7.52E-01 |
| Alkaline phosphatase | 30610 | 3.944e-04 | 2.610e-03 | 8.80E-01 |
| Glycated haemoglobin (HbA1c) | 30750 | -7.137e-05 | 1.450e-03 | 9.61E-01 |
| Red blood cell (erythrocyte) distribution width | 30070 | 3.797e-02 | 2.549e-03 | 3.52E-50 |
| Platelet crit | 30090 | 2.647e-02 | 2.543e-03 | 2.27E-25 |
| Mean sphered cell volume | 30270 | 2.178e-02 | 2.610e-03 | 7.30E-17 |
| Haemoglobin concentration | 30020 | -1.292e-02 | 1.993e-03 | 9.08E-11 |
| Mean corpuscular haemoglobin concentration | 30060 | -1.443e-02 | 2.315e-03 | 4.58E-10 |
| Leg fat percentage (left) | 23115 | -7.269e-03 | 1.310e-03 | 2.85E-08 |
| Body fat percentage | 23099 | -8.893e-03 | 1.609e-03 | 3.23E-08 |
| Arm fat percentage (left) | 23123 | -8.313e-03 | 1.604e-03 | 2.18E-07 |
| Leg fat percentage (right) | 23111 | -6.804e-03 | 1.327e-03 | 2.92E-07 |
| Arm fat percentage (right) | 23119 | -8.175e-03 | 1.608e-03 | 3.71E-07 |
| Mean reticulocyte volume | 30260 | 1.224e-02 | 2.590e-03 | 2.31E-06 |
| Body mass index (BMI) | 23104 | -1.215e-02 | 2.572e-03 | 2.31E-06 |
| Body mass index (BMI) | 21001 | -1.198e-02 | 2.536e-03 | 2.33E-06 |
| Whole body fat mass | 23100 | -1.191e-02 | 2.537e-03 | 2.68E-06 |
| Arm fat mass (right) | 23120 | -1.131e-02 | 2.487e-03 | 5.39E-06 |
| Arm fat mass (left) | 23124 | -1.123e-02 | 2.516e-03 | 8.09E-06 |
| Leg fat mass (left) | 23116 | -7.364e-03 | 1.679e-03 | 1.16E-05 |
| Trunk fat mass | 23128 | -1.122e-02 | 2.564e-03 | 1.21E-05 |
| Leg fat mass (right) | 23112 | -7.386e-03 | 1.697e-03 | 1.35E-05 |
| Reticulocyte count | 30250 | -1.103e-02 | 2.563e-03 | 1.68E-05 |
| Comparative height size at age 10 | 1697 | 7.610e-03 | 1.784e-03 | 1.99E-05 |
| Mean corpuscular haemoglobin | 30050 | -1.076e-02 | 2.571e-03 | 2.84E-05 |
| Haematocrit percentage | 30030 | -8.281e-03 | 2.057e-03 | 5.66E-05 |
| Reticulocyte percentage | 30240 | -9.691e-03 | 2.589e-03 | 1.82E-04 |
| Hip circumference | 49 | -8.957e-03 | 2.571e-03 | 4.94E-04 |
| Red blood cell (erythrocyte) count | 30010 | -7.445e-03 | 2.226e-03 | 8.26E-04 |
| High light scatter reticulocyte count | 30300 | -8.346e-03 | 2.592e-03 | 1.28E-03 |
| Sleep duration | 1160 | 5.159e-03 | 1.677e-03 | 2.10E-03 |
| Waist circumference | 48 | -5.742e-03 | 1.886e-03 | 2.33E-03 |
| Mean carotid IMT (intima-medial thickness) at 210 degrees | 22677 | 2.721e-02 | 9.005e-03 | 2.51E-03 |
| Age at recruitment | 21022 | -1.232e-03 | 4.267e-04 | 3.88E-03 |
| Minimum carotid IMT (intima-medial thickness) at 210 degrees | 22676 | 2.572e-02 | 9.216e-03 | 5.26E-03 |
| High light scatter reticulocyte percentage | 30290 | -7.000e-03 | 2.611e-03 | 7.34E-03 |
| Mean carotid IMT (intima-medial thickness) at 120 degrees | 22671 | 2.301e-02 | 8.920e-03 | 9.89E-03 |
| Weight | 21002 | -5.842e-03 | 2.289e-03 | 1.07E-02 |
| Maximum carotid IMT (intima-medial thickness) at 240 degrees | 22681 | 2.319e-02 | 9.117e-03 | 1.10E-02 |
| Weight | 23098 | -5.752e-03 | 2.285e-03 | 1.18E-02 |
| Sleeplessness / insomnia | 1200 | -3.763e-03 | 1.537e-03 | 1.44E-02 |
| Maximum carotid IMT (intima-medial thickness) at 210 degrees | 22678 | 2.199e-02 | 9.042e-03 | 1.50E-02 |
| Minimum carotid IMT (intima-medial thickness) at 120 degrees | 22670 | 2.101e-02 | 9.159e-03 | 2.18E-02 |
| Maximum carotid IMT (intima-medial thickness) at 120 degrees | 22672 | 2.023e-02 | 8.974e-03 | 2.42E-02 |
| Minimum carotid IMT (intima-medial thickness) at 150 degrees | 22673 | 1.985e-02 | 9.135e-03 | 2.98E-02 |
| Eosinophill percentage | 30210 | 5.342e-03 | 2.589e-03 | 3.91E-02 |
| Mean carotid IMT (intima-medial thickness) at 150 degrees | 22674 | 1.792e-02 | 9.003e-03 | 4.66E-02 |
| Maximum carotid IMT (intima-medial thickness) at 150 degrees | 22675 | 1.769e-02 | 9.047e-03 | 5.05E-02 |
| Genetic principal components | 22009 | 2.748e-02 | 1.406e-02 | 5.06E-02 |
| Overall health rating | 2178 | -3.031e-03 | 1.579e-03 | 5.48E-02 |
| Neuroticism score | 20127 | -4.962e-03 | 2.587e-03 | 5.51E-02 |
| Mean carotid IMT (intima-medial thickness) at 240 degrees | 22680 | 1.740e-02 | 9.077e-03 | 5.52E-02 |
| White blood cell (leukocyte) count | 30000 | -3.879e-03 | 2.165e-03 | 7.32E-02 |
| Sitting height | 20015 | 1.584e-02 | 8.849e-03 | 7.34E-02 |
| Trunk fat-free mass | 23129 | 2.952e-03 | 1.671e-03 | 7.73E-02 |
| Age diabetes diagnosed | 2976 | -1.319e-02 | 7.782e-03 | 9.01E-02 |
| Trunk predicted mass | 23130 | 2.789e-03 | 1.650e-03 | 9.10E-02 |
| Neutrophill count | 30140 | -3.573e-03 | 2.154e-03 | 9.72E-02 |
| Eosinophill count | 30150 | 4.073e-03 | 2.576e-03 | 1.14E-01 |
| Basophill percentage | 30220 | 3.730e-03 | 2.362e-03 | 1.14E-01 |
| QRS duration | 12340 | -9.527e-03 | 7.455e-03 | 2.01E-01 |
| P duration | 12338 | 1.034e-02 | 8.129e-03 | 2.03E-01 |
| Microalbumin in urine | 30500 | 5.115e-03 | 4.025e-03 | 2.04E-01 |
| Minimum carotid IMT (intima-medial thickness) at 240 degrees | 22679 | 1.177e-02 | 9.264e-03 | 2.04E-01 |
| Systolic blood pressure, automated reading | 4080 | 2.924e-03 | 2.345e-03 | 2.13E-01 |
| Pulse rate | 4194 | -4.823e-03 | 3.929e-03 | 2.20E-01 |
| Pulse rate, automated reading | 102 | -2.984e-03 | 2.534e-03 | 2.39E-01 |
| Lymphocyte count | 30120 | -2.933e-03 | 2.568e-03 | 2.53E-01 |
| Fat | 100004 | 5.945e-03 | 5.726e-03 | 2.99E-01 |
| Monocyte count | 30130 | -2.625e-03 | 2.552e-03 | 3.04E-01 |
| Age high blood pressure diagnosed | 2966 | -3.475e-03 | 3.543e-03 | 3.27E-01 |
| Whole body fat-free mass | 23101 | 1.606e-03 | 1.661e-03 | 3.34E-01 |
| Age deep-vein thrombosis (DVT, blood clot in leg) diagnosed | 4012 | 1.369e-02 | 1.454e-02 | 3.46E-01 |
| Recent feelings of tiredness or low energy | 20519 | 2.798e-03 | 3.233e-03 | 3.87E-01 |
| Birth weight | 20022 | 2.688e-03 | 3.110e-03 | 3.88E-01 |
| Trouble falling or staying asleep, or sleeping too much | 20517 | 3.038e-03 | 3.566e-03 | 3.94E-01 |
| Diastolic blood pressure, automated reading | 4079 | 2.092e-03 | 2.465e-03 | 3.96E-01 |
| Whole body water mass | 23102 | 1.314e-03 | 1.653e-03 | 4.27E-01 |
| Prospective memory result | 20018 | 1.506e-03 | 1.921e-03 | 4.33E-01 |
| Creatinine (enzymatic) in urine | 30510 | 1.695e-03 | 2.209e-03 | 4.43E-01 |
| Alcohol | 100022 | 3.495e-03 | 4.719e-03 | 4.59E-01 |
| Getting up in morning | 1170 | -1.196e-03 | 1.632e-03 | 4.64E-01 |
| Time spent doing vigorous physical activity | 104900 | -5.933e-03 | 8.102e-03 | 4.64E-01 |
| Morning/evening person (chronotype) | 1180 | 1.738e-03 | 2.375e-03 | 4.64E-01 |
| Age heart attack diagnosed | 3894 | -9.173e-03 | 1.272e-02 | 4.71E-01 |
| Neutrophill percentage | 30200 | -1.760e-03 | 2.504e-03 | 4.82E-01 |
| Carbohydrate | 100005 | -3.768e-03 | 5.719e-03 | 5.10E-01 |
| Food weight | 100001 | 3.560e-03 | 5.787e-03 | 5.38E-01 |
| Time spent doing moderate physical activity | 104910 | 5.386e-03 | 9.076e-03 | 5.53E-01 |
| Mean corpuscular volume | 30040 | -1.521e-03 | 2.592e-03 | 5.57E-01 |
| Time spent doing light physical activity | 104920 | 3.819e-03 | 6.537e-03 | 5.59E-01 |
| Facial ageing | 1757 | -5.982e-04 | 1.108e-03 | 5.89E-01 |
| Basophill count | 30160 | -9.769e-04 | 1.847e-03 | 5.97E-01 |
| Impedance of arm (right) | 23109 | 9.474e-04 | 1.849e-03 | 6.08E-01 |
| Portion size | 100010 | -1.254e-03 | 2.498e-03 | 6.16E-01 |
| Nap during day | 1190 | -5.983e-04 | 1.274e-03 | 6.39E-01 |
| Monocyte percentage | 30190 | -1.139e-03 | 2.533e-03 | 6.53E-01 |
| Fluid intelligence score | 20016 | -1.641e-03 | 3.929e-03 | 6.76E-01 |
| Impedance of arm (left) | 23110 | 7.569e-04 | 1.819e-03 | 6.77E-01 |
| Ventricular rate | 12336 | -3.223e-03 | 8.023e-03 | 6.88E-01 |
| Arm predicted mass (left) | 23126 | -6.525e-04 | 1.650e-03 | 6.93E-01 |
| Energy | 100002 | 2.119e-03 | 5.634e-03 | 7.07E-01 |
| Arm fat-free mass (right) | 23121 | -5.364e-04 | 1.635e-03 | 7.43E-01 |
| Leg predicted mass (left) | 23118 | -5.001e-04 | 1.682e-03 | 7.66E-01 |
| Past tobacco smoking | 1249 | 8.976e-04 | 3.020e-03 | 7.66E-01 |
| Current tobacco smoking | 1239 | -3.420e-04 | 1.183e-03 | 7.73E-01 |
| Immature reticulocyte fraction | 30280 | 7.103e-04 | 2.505e-03 | 7.77E-01 |
| Leg fat-free mass (left) | 23117 | -4.874e-04 | 1.722e-03 | 7.77E-01 |
| Impedance of leg (right) | 23107 | 6.057e-04 | 2.340e-03 | 7.96E-01 |
| Pulse wave Arterial Stiffness index | 21021 | -8.681e-04 | 3.705e-03 | 8.15E-01 |
| Mean platelet (thrombocyte) volume | 30100 | -2.343e-03 | 1.221e-02 | 8.48E-01 |
| Arm predicted mass (right) | 23122 | 3.020e-04 | 1.619e-03 | 8.52E-01 |
| Impedance of leg (left) | 23108 | -3.913e-04 | 2.381e-03 | 8.70E-01 |
| Age stroke diagnosed | 4056 | -2.149e-03 | 1.315e-02 | 8.70E-01 |
| Leg fat-free mass (right) | 23113 | -2.740e-04 | 1.689e-03 | 8.71E-01 |
| Leg predicted mass (right) | 23114 | -2.619e-04 | 1.686e-03 | 8.77E-01 |
| Protein | 100003 | -6.968e-04 | 5.614e-03 | 9.01E-01 |
| Weight change compared with 1 year ago | 2306 | -1.207e-04 | 1.437e-03 | 9.33E-01 |
| Basal metabolic rate | 23105 | 1.384e-04 | 1.737e-03 | 9.37E-01 |
| Arm fat-free mass (left) | 23125 | -7.564e-05 | 1.655e-03 | 9.64E-01 |
| Comparative body size at age 10 | 1687 | 5.754e-05 | 1.457e-03 | 9.69E-01 |
| Lymphocyte percentage | 30180 | 8.811e-05 | 2.484e-03 | 9.72E-01 |
| Anterior thigh lean muscle volume (left) | 22405 | -3.425e-04 | 1.246e-02 | 9.78E-01 |
| Impedance of whole body | 23106 | -4.692e-05 | 1.984e-03 | 9.81E-01 |
| Anterior thigh lean muscle volume (right) | 22403 | -2.882e-04 | 1.242e-02 | 9.82E-01 |
